# Single‐Cell Transcriptomic Atlas of Peripheral Blood Reveals B‐Cell‐Driven Signature Predictive of Acute Pancreatitis Severity

**DOI:** 10.1002/mco2.70350

**Published:** 2025-09-14

**Authors:** Rongli Xie, Guohui Xiao, Kaige Yang, Xiaofeng Wang, Cong Chen, Min Ding, Tong Zhou, Rajarshi Mukherjee, Robert Sutton, Erzhen Chen, Ying Chen, Wei Huang, Dan Xu, Jian Fei

**Affiliations:** ^1^ Department of General Surgery RuiJin Hospital Lu Wan Branch Shanghai Jiao Tong University School of Medicine Shanghai China; ^2^ Department of General Surgery Ruijin Hospital Shanghai Jiao Tong University School of Medicine Shanghai China; ^3^ Department of Emergency Ruijin Hospital Shanghai Jiao Tong University School of Medicine Shanghai China; ^4^ Department of Pediatrics Ruijin Hospital Shanghai Jiao Tong University School of Medicine Shanghai China; ^5^ Liverpool EmerGenT Academy Department of Emergency General and Major Trauma Surgery Aintree University Hospital Liverpool University Hospitals NHS Foundation Trust Liverpool UK; ^6^ Institute of Systems Molecular & Integrative Biology University of Liverpool Liverpool UK; ^7^ West China Centre of Excellence for Pancreatitis Institute of Integrated Traditional Chinese and Western Medicine West China‐Liverpool Biomedical Research Centre West China Hospital Sichuan University Chengdu China; ^8^ West China Biobank West China Hospital Sichuan University Chengdu China

**Keywords:** acute pancreatitis, B cells, machine learning, molecular biomarker, severity prediction

## Abstract

Effective early prediction of acute pancreatitis (AP) severity remains an unmet clinical need due to limited molecular characterization of systemic immune responses. We performed integrated single‐cell RNA sequencing with T‐ and B‐cell receptor profiling on peripheral blood mononuclear cells from AP patients (*n* = 7) at days 1, 3, and 7 after admission. Immune landscape analysis revealed marked inter‐patient heterogeneity, with a distinct expansion of MZB1‐expressing plasma cells that were strongly associated with complicated AP and recovery. Functional validation in an independent cohort (*n* = 14) confirmed disease‐associated plasma cell markers, alongside altered serum immunoglobulin and cytokine profiles (*n* = 32). From these findings, we established a nine‐gene B‐cell‐derived transcriptomic signature (*S100A8*, *DUSP1*, *JUN*, *HBA2*, *FOS*, *CYBA*, *JUNB*, *S100A9*, and *WDR83OS*) predictive of AP severity. This model demonstrated high discriminative performance in internal validation (*n* = 114; AUROC > 0.95, superior to standard clinical scoring systems), and sustained accuracy in external validation cohorts of AP (*n* = 87) and AP combined with non‐AP sepsis (*n* = 174) for predicting persistent organ failure. Our study identifies a mechanistic and predictive role for MZB1⁺ plasma cells in AP pathogenesis, offering a novel immune‐based stratification strategy with potential for precision clinical management.

## Graphical abstract

Effective early prediction of AP severity remains an unmet clinical need. By integrating single‐cell transcriptomics with machine learning, we systematically profiled the peripheral immune landscape in AP. Our analysis uncovered a novel B‐cell driven, nine‐gene signature that is strongly associated with disease severity and was successfully translated into a predictive model with high diagnostic performance.

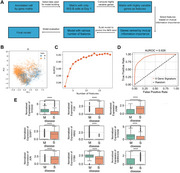

## 1 INTRODUCTION

Acute pancreatitis (AP) is an inflammatory disease initiating from the exocrine pancreas, which sometimes can cause pancreatic necrosis, systemic inflammation, organ failure, and infection, carrying substantial morbidity and mortality [1]. It is one of the most common digestive diseases that causing more than 100 visits to emergence room in the United States per 100,000 persons annually [2] with increasing global incidence over decades [3] and in projection [4]. Biliary factors, alcohol excess, and hypertriglyceridemia are among the major etiologies alone or in combination for this disease [1]. At circa of 15%–25% of patients will develop complications pertaining to pancreas and distal organ systems [5]. Persistent organ failure or multiple organ dysfunction syndrome (MODS) is now recognized as the hallmark of severe AP [6]. This condition carries a high mortality risk, primarily due to systemic complications such as respiratory, circulatory, and/or renal failure that occur at disease early stage [7, 8]. All local complications are principally responsible for higher morbidity secondary to moderately severe AP [6] and when pancreatic necrosis becomes infected, drainage and necrosectomy procedures are inevitable [9]. So far, no internationally licensed effective drugs are available for this potentially devastating disease [1].

Early and accurately identify high risk patients who will develop complications on admission and within 24 h is crucial to guide prompt clinical decisions for triage, interventions, and transfer for AP management. To fulfil this aim, clinical severity scores such as Systemic Inflammatory Response Syndrome (SIRS), Bedside Index for Severity in Acute Pancreatitis (BISAP), Acute Physiology, and Chronic Health Examination II (APACHE II), modified Marshall, and Computerized Tomography Severity Index (CTSI) have been established. Recently, artificial intelligence has been employed to improve predictive accuracy for AP severity. Prospective, multiple center studies have demonstrated that XGBoost machine learning algorithm using five routine clinical indices (with glucose) or six conventional biomarkers yielded an area under the receiver operating characteristic curve (AUROC) of 0.81 (*n* = 4727) and 0.757 (*n* = 2387) for persistent organ failure and pancreatic necrosis, respectively [10, 11]. However, these clinical severity scores, routine biochemical parameters, and machine learning algorithms have reached their limit for early prediction of AP complications and do not potently aide clinical decision [12].

To bridge this gap, researchers have begun to explore the use of immune cell markers along with molecular alterations to predict AP severity. Different circulating monocytes [13] and T‐cell subsets [14] have been mostly studied with some of them have demonstrated to have good predictive value at disease early stage. The studies of B cells are in paucity but collectively demonstrating that increased number of B cells with suppressed function and decreased regulatory B cells are observed in AP with complications; furthermore, serum levels of IgM and IgG are significantly reduced in patients with infectious complications [14]. Recently, circulating immune cell transcriptomic signatures (*S100A8*, *S100A9*, *MMP25*, and *MT‐ND4L* [15]; *CBLB*, *JADE2*, and RNF144A [16]) have been found to predict AP with complications. Furthermore, immunogenic cell death‐associated genes (*LY96*, *BCL2*, and *IFNGR1*) have been identified as early predictive biomarkers for persistent organ failure [17]. Despite these findings, the precise role and predictive value of these immune cells from peripheral blood mononuclear cells (PBMCs) during AP severity progression and subsequent resolution remain largely unexplored.

In light of these considerations, our study employed single‐cell RNA sequencing (scRNA‐seq) combined with single‐cell sequencing of T‐cell receptor (scTCR‐seq) and B‐cell receptor (scBCR‐seq) to profile adaptive immune cells from PBMCs of AP patients. Our results reveal the dynamic adaptive immune characteristics and demonstrate that increase in plasma cell populations and diversity of TCR/BCR are key indicators of severity progression and recovery. By integrating the comprehensive single‐cell atlas derived from B cells on admission, we developed and validated a machine learning model using nine‐gene signature that effectively predicted AP with complications or persistent organ failure. These findings not only enhance our understanding of the pathophysiology of AP with complicated clinical disease course but also provide potential therapeutic targets for tackling its progression. Furthermore, since the admission B‐cell‐derived transcriptomic signature had significant higher predictive AUROC than SIRS, BISAP, APACHE II, modified Marshall score, and CTSI, it holds the promise for clinical translation as an early predictive marker.

## 2 RESULTS

### 2.1 Single‐Cell Transcriptomics of PBMCs Reveals Heterogeneous Immune Responses

**FIGURE 1:**
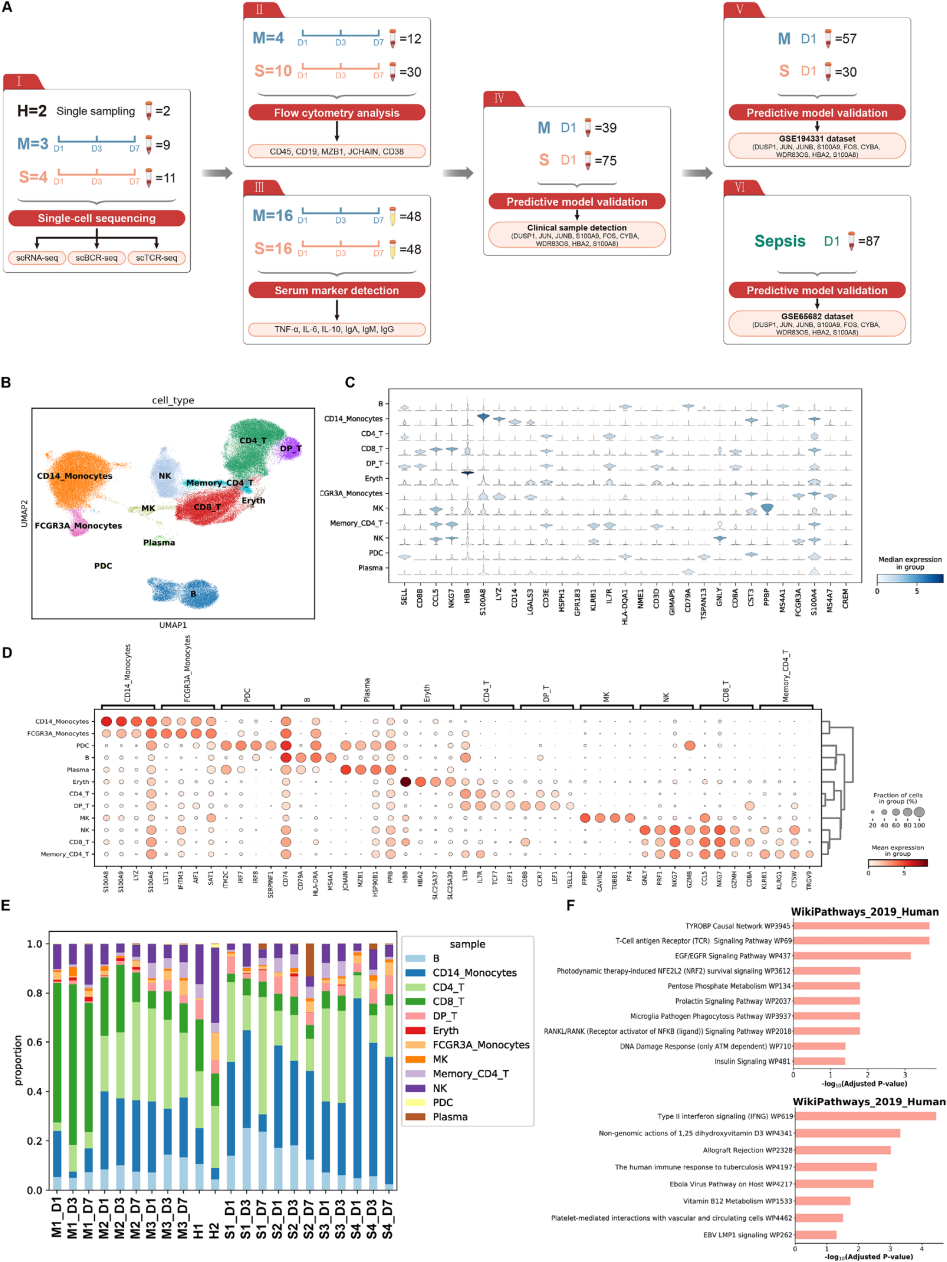
Flow chart of study design and immune cell atlas in the circulation of patients with AP. (A) Schematic diagram of clinical sample collection and testing in different cohorts. The analysis of scRNA‐seq includes PBMC samples from two healthy donors and seven AP patients (three uncomplicated and four complicated). (B) Clustering and annotation of 12 cell types demonstrated in 2D UMAP. (C) Expression levels of known marker genes among annotated cell types. (D) Top DEGs identified for each cell type. Colors reflected gene expression levels. Circle sizes reflected proportions of cells with nonzero gene expression level. (E) Proportion of each cell type per sample. Samples were labeled as “disease,” “patient,” and “days.” M1_1 was the first M case collected at day 1. H1 and H2 were two healthy donors (they only had blood withdrawn for once). (F) Pathway enrichment of DEGs in CD14^+^ monocytes in complicated (top) and uncomplicated (bottom) cases. Human WikiPathways (2019 version) was selected as reference database. DEGs, differentially expressed genes.

To characterize immune response landscape reflecting longitudinal trajectory of AP clinical course, we initially built a transcriptomic atlas using scRNA‐seq of PBMCs collected from patients and healthy volunteers, followed by scTCR‐seq, scBCR‐seq (Figure 1AI), functional validations (Figure 1AII,III), internal prediction model developing and validation (Figure 1AIV) as well as external validations (Figure 1AV,VI). For single‐cell transcriptomic atlas, the PBMCs were collected at days 1, 3, and 7 after admission from four moderately severe and severe (“S,” complicated) and three mild (“M,” uncomplicated) AP defined by the revised Atlanta classification [6]. After quality control, 102,042 cells were retained with medians of 1340 genes and 3565 transcripts were detected per cell. After batch correction, the cells were grouped into 14 initial clusters and annotated to 12 cell types (Figure S1A,B), including antibody‐secreting plasma B cell (thereafter referred as “plasma cells”) and non‐plasma cells, CD14^+^ monocytes, FCGR3A^+^ monocytes (CD16^+^ subtype monocytes), CD4^+^ T cells, CD8^+^ T cells, CD4 and CD8 double positive T cells (DP T cells), memory CD4^+^ T cells, erythrocytes, megakaryocytes, natural killer cells, and plasmacytoid dendritic cells demonstrated by the UMAP (Uniform Manifold Approximation and Projection) plot (Figure 1B). Several known immune cell markers were used to annotate the clusters in our data, such as *CD14* for CD14^+^ monocytes and CD79A for B cells (Figure 1C). Thereafter, differential expression analysis was conducted to unveil the top markers for each annotated cell types and their specific expression pattern. Moreover, hierarchical clustering was performed on cells based on their coordinates in reduced dimensions. These results reveal the expected clustering patterns and further consolidate the reliability of our annotation (Figure 1D). Interestingly, the cell–cell interaction analysis showed that the immune cells would interact with each other through ligand–receptor pairs; among them, it was demonstrated that CD8^+^ T cells and DP T cells had the most active overall interaction (Figure S2). Then the alteration of immune cell population for each sample at individual designated sampling time node was further investigated, depicting a highly complex and variable immune responses between complicated and uncomplicated patients during disease trajectory (Figure 1E).

Subsequently, a pathway analysis was undertaken to explore the functions of differentially expressed genes (DEGs) of CD14^+^ monocytes, the proportions of which were observably differed between complicated and uncomplicated cases. In the complicated group, CD14^+^ monocyte including TYROBP (transmembrane immune signaling adaptor) causal network, TCR, EGF/EGFR, NRF2, pentose phosphate metabolism, prolactin, microglia pathogen phagocytosis, RANKL/RANK (receptor activator of NF‐κB [ligand]), DNA damage response, and insulin signaling pathways was found to be the highly enriched pathways (Figure 1F, upper panel). In the uncomplicated group, type II interferon signaling, non‐genomic action of 1,25 dihydroxyvitamin D3, vitamin B12, and platelet‐mediated interactions with vascular and circulating cells were highly enriched in CD14^+^ monocytes (Figure 1F, lower panel).

To validate immune cell subtype dynamics across different severity categories, we established mouse AP models induced by cerulein (CER‐AP) or cerulein with lipopolysaccharide (CER/LPS‐AP) [18]. Flow cytometry analysis of pancreata and PBMCs collected at 12 h after disease induction reveal distinct immune cell redistribution (Figure S3A). Macrophages were elevated in the pancreas and PBMCs of both CER‐AP and CER/LPS‐AP (Figure S3B). While Ly6C^high^ macrophages (corresponding to human CD14^+^ monocytes) were elevated in the PBMCs of both CER‐AP and CER/LPS‐AP, the parallel increase in the pancreas was only observed in CER/LPS‐AP, indicating expanded Ly6C^high^ macrophages infiltration in more severe disease phenotype. Ly6C^low^ macrophages (human CD16^+^ monocytes) were increased in the pancreas and PBMCs of both CER‐AP and CER/LPS‐AP (Figure S3C). Interestingly, plasma cells were significantly elevated in pancreas, while the number of B cells were reduced in the PBMCS of CER/LPS‐AP (Figure S3D,E). No such changes were noted in the CER‐AP. The pancreatic‐specific accumulation of plasma cells in more severe experimental AP phenotype highlighted their potential role in tissue‐specific immune responses and positioned them as candidate prognostic biomarkers.

#### 2.1.1 Single‐Cell TCR Profiling Reveals Correlation Between TCR Diversity and Disease Severity

**FIGURE 2:**
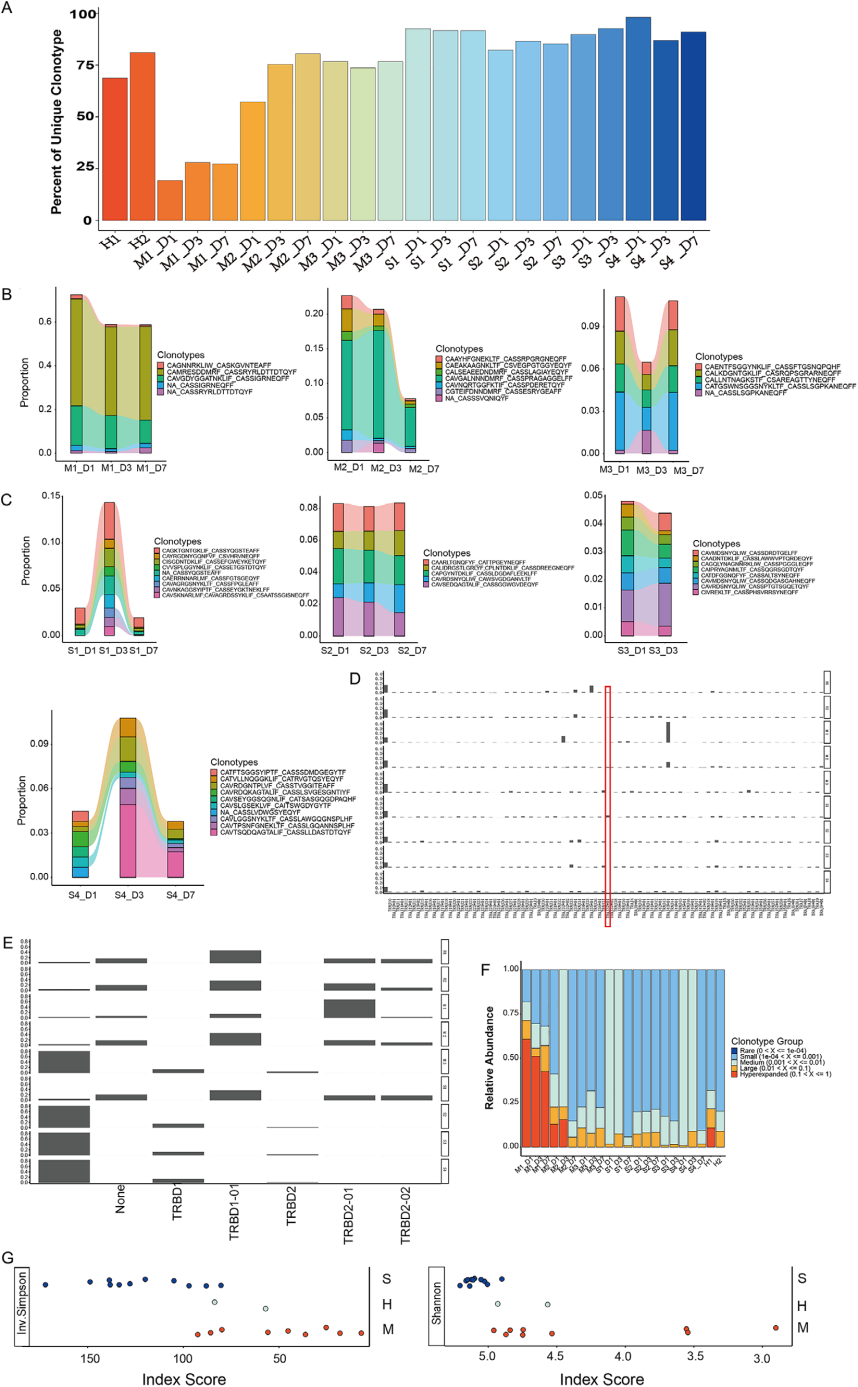
Characterization of scTCR‐seq for immune cells in the circulation. (A) Quantitation of unique clonotypes in each sample. Data derived from scTCR‐seq of PBMCs from seven AP patients (three uncomplicated and four complicated). (B–D) Comparison of top 10 clonotypes at each sampling day for uncomplicated and complicated cases. (E) V gene usage on TCR‐α chain of each patient. (F) D gene usage on TCR‐β chain of each patient. (F) Clonal space homeostasis analysis of each sample. (G) Diversity analysis using Shannon and inverse Simpson. TCR, T‐cell receptor.

We analyzed the clonotypes using the V(D)J genes encoding the TCR and the nucleotide sequence of the CDR3 region for the TCR‐α and ‐β chains using scTCR‐seq data from the PBMCs (Figure 2A, Figure S4A). Our findings reveal their dynamics in the TCR repertoire, indicating complicated AP patients had relatively more unique clonotypes than uncomplicated patients or healthy volunteers. Then, we examined the dynamic changes of the top 10 clonotypes in each sample. In all uncomplicated patients, the proportions of the common clonotypes were slightly decreased during recovery at day 3; they continued to decrease in cases M1 and M2, while were back to the initial levels in M3 (a recurrent case) at day 7 (Figure 2B). In complicated patients, the proportions of the common clonotypes were increased in cases S1 and S4, and recovered at day 7; while in cases S2 and S3, they remained unchanged from day 1 to 7 or to discharge at day 3, respectively (Figure 2C). These changes were in accord with clinical manifestations of respective donor patients. In cases S1 and S4, the SIRS and organ dysfunction persisted at day 3 and only alleviated at day 7, while in cases S2 and S3, SIRS and organ dysfunction resolved at day 3. The results suggest the distinctive and dynamic patterns of the TCR repertoire of AP patients between different severity and during the disease progression and recovery.

We then examined *VDJ* gene usage in the TCR‐α and ‐β chains and observed relatively higher TRAJ37*01 usage in case S1 (Figure 2D); there were similar levels of *D* gene usage among other complicated cases as well as case M3 (Figure 2E). Subsequently, the relative space occupied by clones at specific proportions was examined. Cases M1 and M2 showed a large proportion of hyperexpanded clonotypes (0.1 < frequency ≤ 1). Case M3 and all complicated cases presented with a large proportion of clonotypes belonging to a small group (10^−4^ < frequency ≤ 10^−3^) (Figure 2F, Figure S4B). Furthermore, it was shown that complicated cases had more profound clonotype diversity than those of uncomplicated cases or healthy volunteers, illustrated by Shannon and inverse Simpson indices (Figure 2G).

#### 2.1.2 Single‐Cell BCR Profiling Unravels B‐Cell Diversity Reflecting Disease Severity

**FIGURE 3:**
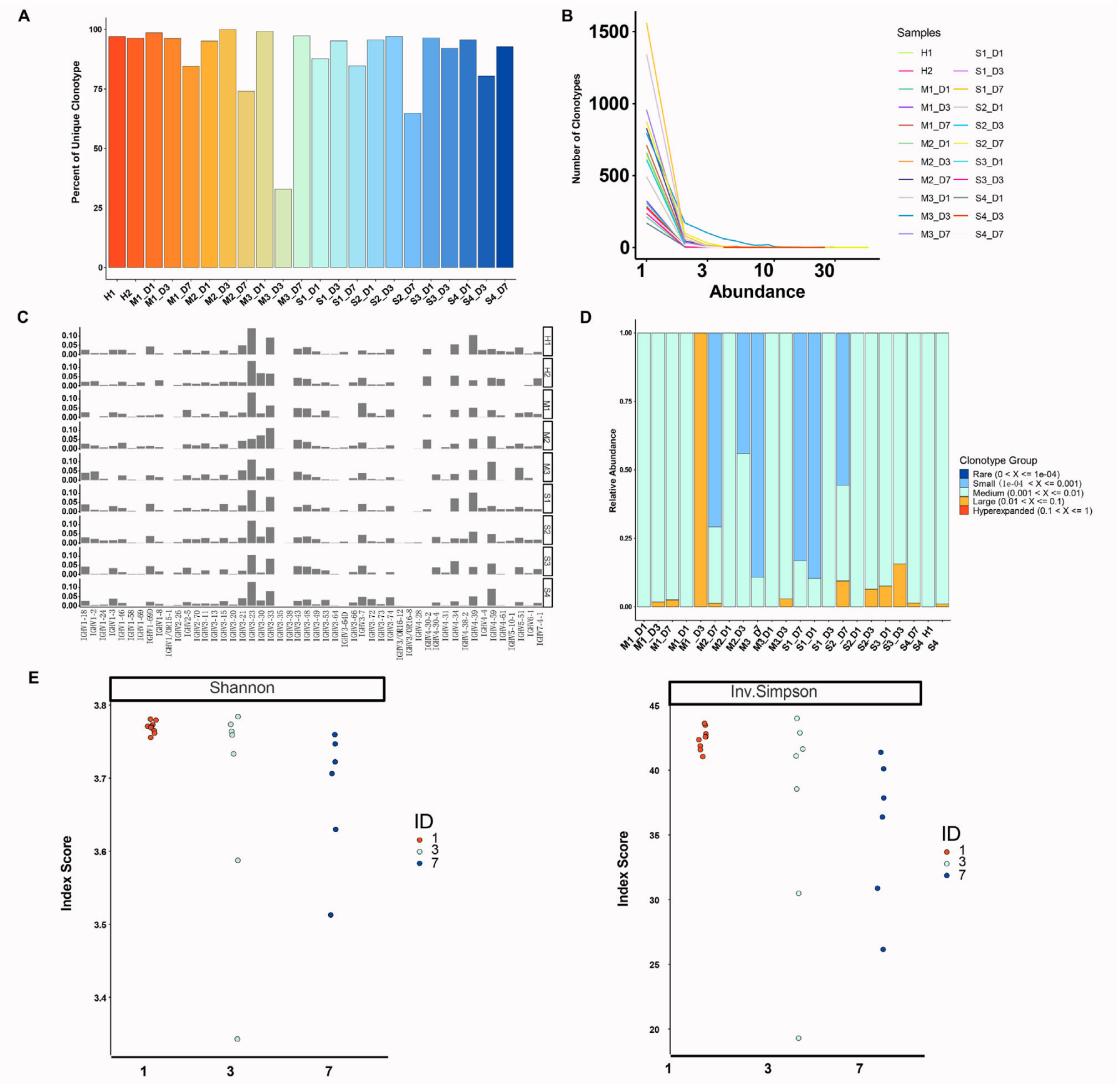
Characterization of scBCR‐seq for immune cells in the circulation. (A) Quantitation of unique clonotypes in each sample. Data derived from scBCR‐seq of PBMCs from seven AP patients (three uncomplicated and four complicated). (B) Relative distributions of clonotypes by abundance in each sample. (C) V gene usage profile on IGH chain of each patient. (D) Clonal space homeostasis analysis of each sample. (E) Diversity analysis using Shannon and inverse Simpson for sampling days and conditions. BCR, B‐cell receptor; IGH, immunoglobulin heavy chain.

To investigate the immunological characteristics of B cells, we used scBCR‐seq data from the PBMCs. By identifying clonotypes using the V(D)J genes encoding the BCR and the nucleotide sequence of the CDR3 region for both immunoglobulin heavy (IGH) and light chains, we found that for complicated cases, the proportions of unique clonotypes were similar for both BCR and TCR. However, the percentages of unique clonotype of BCR for uncomplicated cases were relatively higher than those of TCR (Figures 2A and 3A, Figure S5A). Then we examined the relative clonotype abundance and distribution for each patient. Two complicated cases S1 and S2 displayed substantially higher clonotype diversity than other patients. This discrepancy might affect complicated cases in terms of immune repertoire diversity (Figure 3B). *VDJ* gene usage analysis found that case S2 alone used IGHV4‐38‐2, while cases S2, S3, S4, and M3 used IGHV4‐30‐4 (Figure 3C). Examination of the proportions of clonal space occupied by the ranked clonotype groups revealed that the hyperexpanded group was absent in all AP patients, in contrast to the TCR repertoire (Figure 3D, Figure S5B). In addition, Shannon and inverse Simpson indices analysis demonstrated PBMCs drawn at days 1 and 3 had higher clonotype diversity scores than those taken at day 7 (Figure 3E). As a result, the complicated cases were featured by more unique BCR clonotypes than the uncomplicated cases. Furthermore, the clonotype diversity was decreased during AP recovery.

#### 2.1.3 Single‐Cell Transcriptomics Integrated With TCR/BCR Repertoires Elucidate Severity‐Associated Immune Cell Characteristics

**FIGURE 4:**
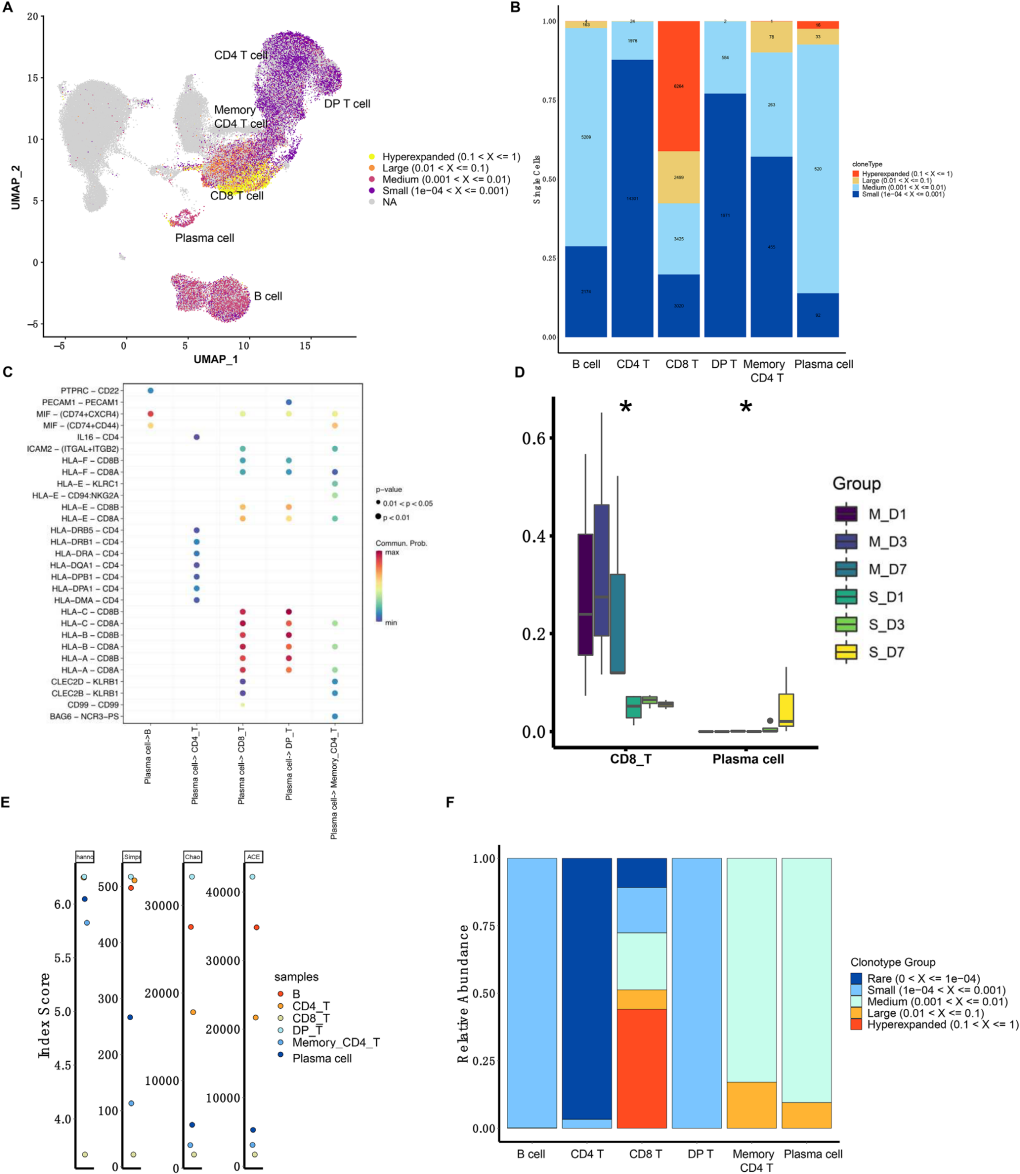
Integration analysis of scRNA‐seq, scTCR‐seq, and scBCR‐seq. (A) UMAP of clonotype distributions in T cells, B cells, and their subtypes. Data derived from scRNA‐seq of PBMCs from seven AP patients (three uncomplicated and four complicated). (B) Numbers of cells by type assigned to specific frequency ranges. (C) Ligand‐receptor pairs that played crucial roles in TCR‐BCR related cell–cell interaction maps. (D) Overall CD8^+^ T and plasma cell proportion changes among patients of the uncomplicated and complicated groups. (E) Diversity analysis using Shannon, inverse Simpson, and Chao indices and ACE for T cells, B cells, and their subtypes. (F) Clonal space homeostasis analysis of T cells, B cells, and their subtypes. ACE, abundance‐based coverage estimator; BCR, B‐cell receptor; TCR, T‐cell receptor.

Subsequently, the clonotype distributions were examined in various immune cell types. Only T cells and B cells exhibited TCR and BCR clonotypes distributions as expected. CD8^+^ T cells had more hyperexpanded clonotypes (0.1 < frequency ≤ 1) than the other T‐cell subtypes (Figure 4A). In addition, CD8^+^ T cells had the highest proportion with hyperexpanded clonotypes (0.1 < frequency ≤ 1), whereas the lowest proportion was assigned to small clonotypes (10^−4^ < frequency ≤ 10^−3^) (Figure 4B). We then examined the cell–cell interactions of plasma cells with other B and T cells. Interestingly, apart from major histocompatibility complex class I genes, multiple critical interactions between plasma cells and CD8^+^ T cells were found and marked by macrophage inhibitory factor (MIF) – (CD74 and chemokine C‐X‐C motif receptor 4 [CXCR4]), intercellular adhesion molecule 2 (ICAM2)—(integrin subunit alpha L [ITGAL] and integrin subunit beta 2 [ITGB2]), C‐type lectin domain family 2 member D and B (CLEC2D and CLEC2B)—killer cell lectin like receptor B1 (KLRB1), and CD99 (Figure 4C). The proportions of CD8^+^ T cells were significantly decreased while plasma cells were significantly increased in complicated patients (Figure 4D). The DP T cells had the highest clonotype diversity score, whereas the CD8^+^ T cells showed the lowest diversity. As expected, non‐plasma cells had higher clonotype diversity scores than plasma cells (Figure 4E) and were assigned to a small clonotype group (10^−4^ < frequency ≤ 10^−3^) (Figure 4F). These results suggest that CD8^+^ T cells were deactivated in complicated patients, while plasma cells were activated and might replenish the immune responses.

#### 2.1.4 Increased Proportion of Plasma Cells Correlates With Disease Prognosis

**FIGURE 5:**
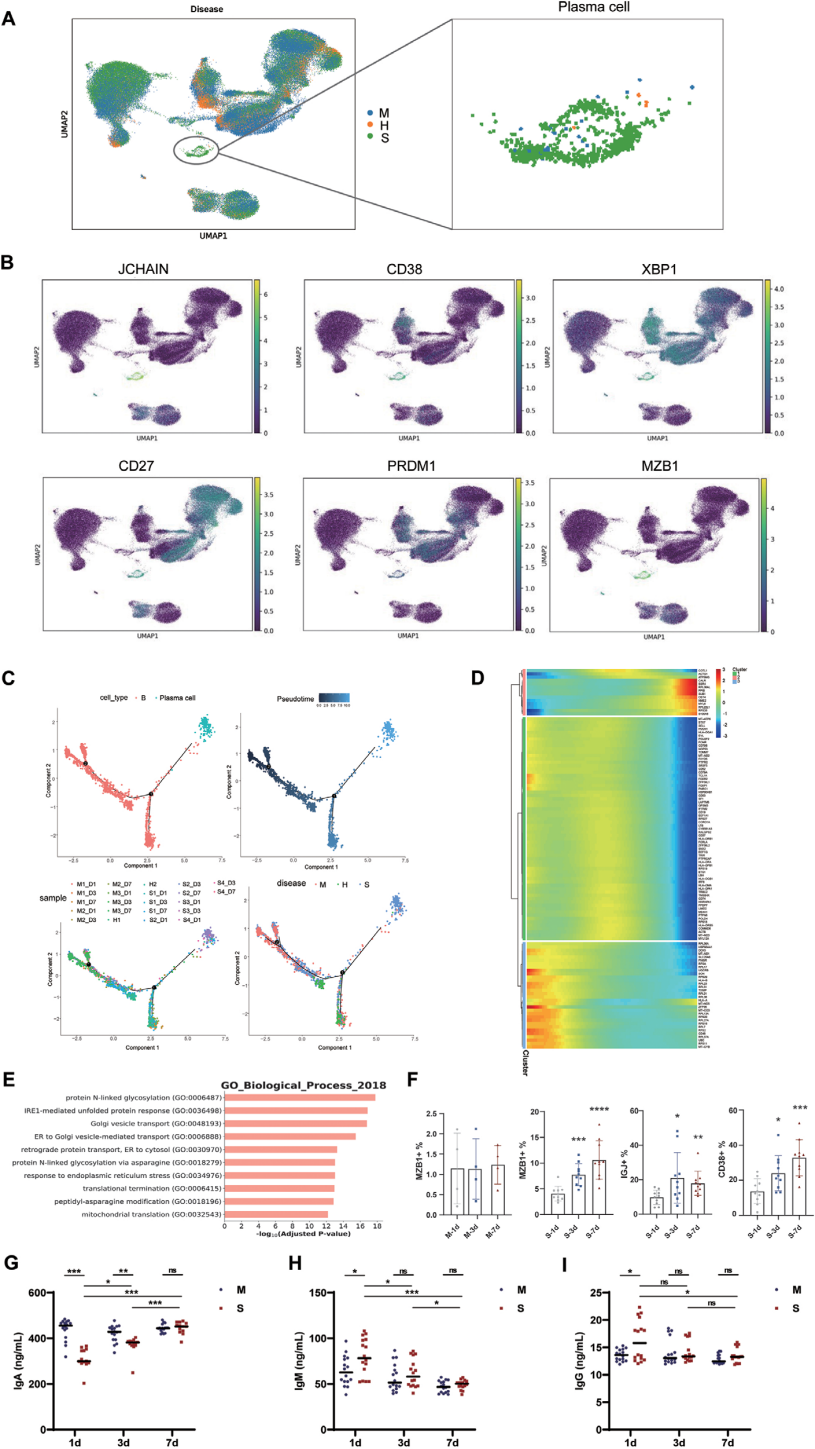
Increased number of plasma cells is associated with complicated AP. (A) PBMC cells demonstrated in 2D UMAP were colored according to disease (uncomplicated/complicated) or healthy control status. (B) The expression profile of plasma cell markers including *JCHAIN*, *CD38*, *XBP1*, *CD27*, *PRDM1*, and *MZB1*. The expression levels of six plasma cell markers were shown on the single cell 2D UMAP. Green color showed higher expression. The gene expression levels were log transformed and normalized at cell level. (C) Pseudotime trajectory reconstruction using Monocle2 for B cells and plasma cells was performed, with cells colored according to their type, progression along the pseudotime axis, sample origin, and disease conditions. (D) Dynamic genes along the B cell and plasma cell pseudotime trajectory were grouped into three clusters based on significant changes in expression. The heatmap illustrates the relative gene expression levels. (E) Gene ontology (GO) enrichment of DEGs in plasma cells. Note that 2018 version of GO biological process was selected as GO term database. (F) Representative flow cytometry quantification of MZB1^+^, IGJ^+^, and CD38^+^ cells from samples of the uncomplicated (*n* = 4) and complicated (*n* = 10) groups at days 1, 3, and 7. (G–I) Representative ELISA quantification of IgA, IgM, and IgG from samples of the uncomplicated (*n* = 16) and complicated (*n* = 16) groups at days 1, 3, and 7. *p < 0.05, **p < 0.01, ***p < 0.001, ****p < 0.0001. DEGs, differentially expressed genes; IGJ, immunoglobulin joining chain; JCHAIN, joining chain of multimeric IgA and IgM; MZB1, marginal zone B and B1 cell‐specific protein; PRDM1, PR/SET domain 1; XBP1, X‐box binding protein 1.

In accord with observations of plasma cells and B cells from CER/LPS‐AP and CER‐AP (Figure S3D,E), only in samples S1_D7, S2_D7, and S4_D3 of our single‐cell atlas, a relatively rare cell population annotated as “plasma cells” was observed (Figure  1B,C, Figure 5A). These cells highly expressing marginal zone B and B1 cell‐specific protein (*MZB1)*, joining chain of multimeric IgA and IgM (*JCHAIN*), and other known plasma cell markers (*CD38*, *XBP1*, *CD27*, and *PRDM1*) are derived from B cells (Figure 5B) [19]. These findings indicate that the number and function of B cells may link with disease recovery in complicated cases.

Pseudotime analysis revealed B cells had quite distinct profiles among the healthy, uncomplicated, and complicated groups. This dynamic process reflected the progressive activation and functional transformation of B cells in response to the evolving disease environment. Moreover, the cells in the transition state between B cells and plasma cells were mostly from AP patients rather than healthy volunteers (Figure 5C). In addition, genes showed dynamics through this trajectory, including known plasma cell marker peptidylprolyl isomerase B (*PPIB*) (Figure 5D). During disease early stage (Figure 5D), cells displayed high expression of B cell‐receptor and antigen‐presentation genes such as “CD79A,” “CD79B,” “HLA‐DRA,” “CD74,” and “PTPRC,” which declined sharply as pseudotime advanced, marking the coordinated shutdown of surface‐receptor signaling that precedes plasma cell commitment. In contrast, genes supporting secretory function and metabolic reprogramming, such as “ACTG1” and “ATP5MG,” were strongly upregulated at the terminal branch. This observation indicates that the B cells from AP patients of the complicated group also had the tendency to develop to plasma cells. Therefore, we hypothesized that the plasma cell proportion might be associated with the prognosis of AP patients.

The gene ontology (GO) enrichment analysis for the DEGs of plasma cells reveal a few terms related to protein synthesis and secretion (Figure 5E). In a separate cohort of 14 AP patients (10 complicated and four uncomplicated) containing 42 PBMC samples (Figure 1AII), the GO enrichment analysis for the DEGs of plasma cells reveal a few terms related to protein synthesis and secretion (Figure 5E). The number of MZB1^+^ cells did not change among any time points (Figure 5F, Figure S6A) in cases of uncomplicated group but showed consistent increase in cases of the complicated group (Figure 5F, Figure S6B). Consistent results of increased plasma cells in the complicated group were verified using another two markers, IGJ (encoded by *JCHAIN*) and CD38 (Figure 5F, Figure S6C,D). These results confirm that that plasma cells were abundantly increased in patients of the complicated group compared with those in the other groups.

In another cohort of 32 AP patients (16 complicated and 16 uncomplicated) containing 96 PBMC samples (Figure 1AIII), the levels of serum immunoglobulins (Figure 5G) and cytokines (Figure S7) were measured. Interestingly, only patients in complicated group had reduced levels of IgA compared with uncomplicated group in which those were unchanged at days 1 and 3; the levels of IgA in the complicated group gradually recovered over the time node and were comparable to uncomplicated group at day 7 (Figure 5G). Both levels of IgM and IgG at day 1 were significantly higher in the complicated group compared with the uncomplicated group, while this trend diminished at days 3 and 7. Both levels of IgM (Figure 5H) and IgG (Figure 5I) gradually decreased over the time node, compared with a relatively stable condition in the uncomplicated group. The levels of tumor necrosis factor (TNF‐α) and interleukin‐6 (IL‐6) were significantly higher in the complicated group than the uncomplicated group at day 1; their levels had a steady decrease over the time node and this trend was more marked in the complicated group. The levels of IL‐10 were comparable between the two groups and were significantly elevated at day 7 compared with days 1 and 3 in the complicated group.

These results suggest that in complicated AP patients, the proportion of plasma cells in PBMCs was gradually increased with disease progression and subsequent resolution. The increased plasma cells may restrain inflammatory response to alleviate AP complications by secreting IgA.

#### 2.1.5 Machine Learning Model Using B‐Cell‐Derived Transcriptomic Signature for Early Severity Prediction

**FIGURE 6:**
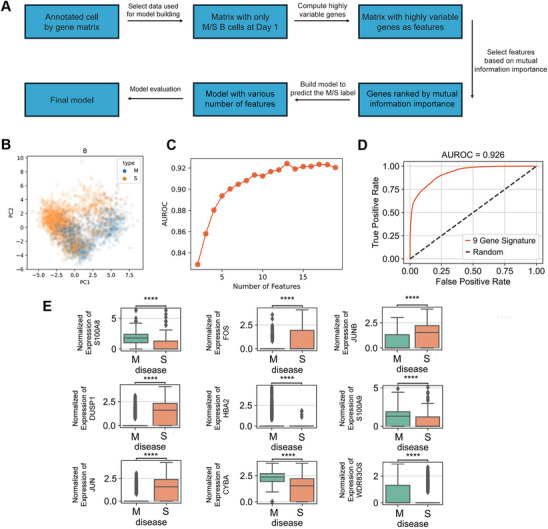
Construction of early predictive model to stratify disease severity based on B cells in the circulation. (A) Overview of model building workflow. (B) 2D embeddings (principal components 1 and 2) of B cells for uncomplicated/complicated at day 1. Cell origins were labeled with different colors depending on disease type. (C) AUROC values for random forest models using different number of genes as input. (D) AUROC curve for final random forest model using the nine genes as input. (E) Expression levels of final nine‐gene signature in B cells at day 1 in patients of uncomplicated (*n* = 3) and complicated (*n* = 4) groups. AUROC, area under the receiver operating characteristic curve.

We used B cells to build an early predictive model aiming to distinguish complicated from uncomplicated cases (Figure 6A). Principal component analysis (PCA) shows the profile of the B cells differed between the two groups (Figure 6B), and random forest classifiers analysis estimates that the AUROC curve values would be stable when the number of features reached 9 (Figure 6C). Using these top nine genes, including *S100A8* (S100 calcium binding protein A8), *DUSP1* (dual specificity phosphatase 1), *JUN* (Jun proto‐oncogene, AP‐1 transcription factor subunit), *HBA2* (hemoglobin subunit alpha 2), *FOS* (Fos proto‐oncogene), *CYBA* (cytochrome b‐245 alpha chain), *JUNB* (JunB proto‐oncogene, activator protein 1 transcription factor subunit), *S100A9*, and *WDR83OS* (WD repeat domain 83 opposite strand), we obtained an effective classifier with AUROC = 0.926 (Figure 6D). Then the mRNA levels of these nine genes in B cells at day 1 were further investigated. The results showed *S100A8*, *HBA2*, *CYBA*, *S100A9*, and *WDR83OS* were significantly decreased, while *DUSP1*, *JUN*, *FOS*, and *JUNB* were obviously increased in B cells in the complicated group compared with those in the uncomplicated group (Figure 6E). Therefore, our nine‐gene predictive model was able to effectively distinguish complicated and uncomplicated cases at day 1, implying the potential value for early predicting AP severity.

To investigate the biological roles of these nine genes, we performed gene enrichment analyses using the GO and Kyoto Encyclopedia of Genes and Genomes (KEGG) pathway databases. Our analysis reveals that the identified genes were significantly associated with multiple pathways and biological processes critical to inflammatory and immune responses (Figure S8). These involved signaling pathways of transcription factor activator protein‐1 complex, IL‐17, integrated stress response, Toll‐like receptor 4 binding, arachidonic acid binding, TNF, and respiratory burst, all well‐established critical cellular events contributing to initiation or aggravation of AP.

The enriched biological functions and pathways provide a robust context that supports the predictive capability of our nine‐gene signature, substantiating our model while also offering potential mechanistic insights for potential therapeutic targets to treat AP complications.

#### 2.1.6 Validation of a Nine‐Gene Signature Machine Learning Model for Early Severity Prediction

**FIGURE 7:**
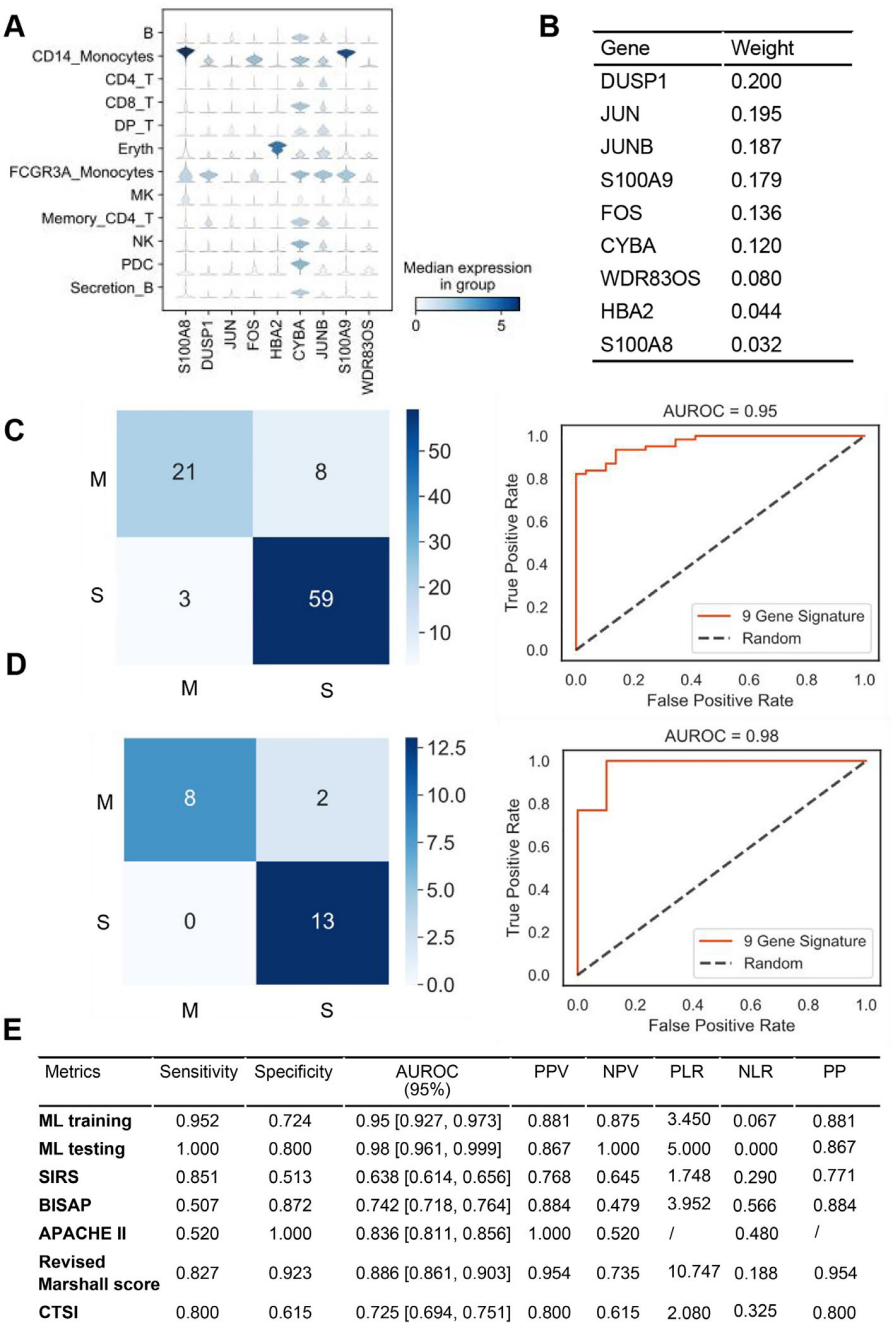
Metrics used to evaluate the random forest classifier in the PBMC samples. (A) Expression profiles of the nine‐gene signature in PBMC subsets at day 1 post admission. (B) Mutual information score of each gene from the nine‐gene signature. (C and D) AUROC for predicting complicated AP. (E) Table showed the sensitivity, specificity, AUROC, PPV, NPV, PLR, NLR, and PP score of the RT‐qPCR‐based machine learning model. AUROC, area under the receiver operating characteristic curve; NLR, negative likelihood ratio; NPV, negative predictive value; PBMC, peripheral blood mononuclear cells; PLR, positive likelihood ratio; PP, post‐test probability; PPV, positive predictive value.

We examined the expressions of nine signature genes in all cell types in AP patients at day 1 using the single‐cell transcriptomic atlas (Figure 1). These genes exhibit moderate expression in B cells when compared with those in other cell types (Figure 7A). Apart from B cells, the expression trends of these genes were also similar in most of the immune cell clusters between complicated and uncomplicated groups (Figure S9). Therefore, we reasoned to apply the nine‐gene signature in the whole PBMCs without sorting of B cells would generalize the machine learning model.

In a cohort of 114 AP patients (complicated: 75, uncomplicated: 39; Table S1), we performed real‐time quantitative reverse transcription PCR (RT‐qPCR) assays on the machine learning‐selected nine genes from PBMCs collected on day 1 (Figure 1AIV; Table S2). The nine‐gene signature contained distinct mutual information scores in classifying the complicated and uncomplicated cases (Figure 7B). Patients in this cohort were randomly split into training (80%, nine = 91) and testing (20%, *n* = 23) datasets. For predicting complicated AP: after 100 training experiments (Table S3), the training set yielded an AUROC of 0.95 (95% confidence interval [CI] 0.927–0.973) with a sensitivity of 0.952 and a specificity of 0.724 (Figure 7C); the testing set achieved an AUROC of 0.98 (95% CI 0.961–0.999) with a sensitivity of 1.000 and a specificity 0.800 (Figure 7D). For predicting persistent organ failure: the training set yielded an AUROC of 0.98 (95% CI 0.961–0.999) with a sensitivity of 0.800 and a specificity of 0.987 (Figure S10A); the testing set achieved an AUROC of 0.97 (95% CI 0.956–0.984) with a sensitivity of 0.750 and a specificity of 1.000 (Figure S10B). All other predictive values for complicated AP and persistent organ failure including positive and negative predictive values, positive and negative likelihood ratios, and post‐test probability are summarized in Figure 7E and Figure S10C. Collectively, the nine‐gene signature machine learning predictive model (AUROC 0.98) outperformed routine clinical severity scoring systems SIRS, BISAP, APACHE II, modified Marshall score, and CTSI which had AUROC ranged from 0.638 to 0.886 for predicting complicated AP.

For external validation of the above predictive model, we employed the GSE194331 dataset [15] that comprises 87 AP patients (complicated: 30, uncomplicated: 57) admitted and with PBMCs collected within 24 h of symptom onset. For predicting complicated AP, this validation set yielded AUROC values of 0.74 (95% CI 0.712–0.768) and 0.77 (95% CI 0.747–0.793) in the training and testing sets, respectively (Figure S11A,B). For predicting persistent organ failure, it had AUROC values of 0.91 (95% CI 0.887–0.933) and 0.85 (95% CI [0.836–0.864]) in the training and testing sets, respectively (Figure S11C,D). Furthermore, by incorporating 87 sepsis patients (non‐AP) from the GSE65682 dataset [20] into the GSE194331 cohort (*n* = 174; AP: 87, sepsis: 87), we demonstrated improved specificity of the predictive model for identifying complicated AP. The Macro‐AUROC was 0.93 (95% CI 0.921–0.939) in the training set and 0.93 (95% CI 0.926–0.934) in the testing set (Figure S12A,B). For predicting persistent organ failure, the Macro‐AUROC was 0.98 (95% CI 0.978–0.982) in the training set and 0.96 (95% CI 0.958–0.962) in the testing set (Figure S12C,D).

These results consistently demonstrate the robustness of our predictive model across different datasets and indicate its prominent value in clinical applications, particularly for predicting persistent organ failure.

## 3 DISCUSSION

In current study, we analyzed single‐cell atlas composed by sequencing data (RNA, TCR, and BCR) of PBMCs collected from AP patients at dynamic time nodes after hospital admission. The single‐cell atlas depicts a heterogeneous immune response between complicated and uncomplicated patients during disease trajectory, particularly those annotated for activation of antibody‐secreting plasma B cells and CD14^+^ monocytes as well as the deactivation of CD8^+^ T cells. It was further noted that the MZB1‐expressing plasma cells were reduced only in AP patients with complications compared with those without and gradually increased with their clinical improvement till hospital discharge. The first decrease and subsequent increase of PBMC plasma cells were tightly mirrored by the serum IgA levels, confirming a major role of plasma cell in association with AP recovery. Most importantly, expanding a nine‐gene transcriptome signature (*S100A8, DUSP1*, *JUN*, *HBA2*, *FOS*, *CYBA*, *JUNB*, *S100A9*, and *WDR83OS*) obtained from B cells to whole immune cells from the PBMCs, the AUROC values remained high across internal and external validation cohorts for predicting AP complications (albeit reduced AUROC for external validation) and persistent organ failure. Of particular note, the nine‐gene transcriptome signature machine learning predictive model had consistent clinical application grade accuracy for predicting persistent organ failure, which is currently identified as the predominate determinant for mortality of this devastating disease.

By integrating the comprehensive single‐cell atlas derived from B cells on admission, we developed and validated a machine learning model using nine‐gene signature that effectively predicted AP with complications or persistent organ failure. These findings not only enhance our understanding of the pathophysiology of AP with complicated clinical disease course but also provide potential therapeutic targets for tackling its progression. Furthermore, since the admission B‐cell‐derived transcriptomic signature had significant higher predictive AUROC than SIRS, BISAP, APACHE II, modified Marshall score, and CTSI, it holds the promise for clinical translation as an early predictive marker.

Our results show that *S100A8*, *HBA2*, *CYBA*, *S100A9*, and *WDR83OS* were significantly decreased, while *DUSP1*, *JUN*, *FOS*, and *JUNB* were obviously increased in B cells of AP patients with complications compared with those without complications. Previous studies demonstrated that *S100A8* and *S100A9* were key components of transcriptomic panel of PBMCs to predict persistent organ failure in AP patients [15]; S100A8 and S100A9 were also increased in the exosomes derived from AP patients with persistent organ failure, which might lead to inflammatory response via NADPH oxidase [21]. In addition, S100A8 and S100A9 were associated with the infiltration of immune cells in the pancreatic tissues of AP patients or experimental models [22]. *S100A9* ^−/−^ mice alleviated AP damage by inhibiting NLRP3 inflammasome activation [23]. *DUSP1*, a member of the mitogen‐activated protein kinase phosphatase family, the deficiency of which increased the expressions of inflammatory factors and aggravated AP severity indices [24]. In contrast with these studies, we found that *S100A8* and *S100A9* were down‐regulated, and *DUSP1* was up‐regulated in B cells of complicated patients compared with those without complications. On the one hand, the previous studies detected the expressions of S100A8, S100A9, and DUSP1 in the blood cells of AP patients or the pancreatic tissues of experimental AP models [15, 21, 24]. We directly measured their levels in the B cell, which might result in the difference. On the other hand, we revealed that the plasma cells, which were derived from the B cells, were gradually increased during AP recovery, indicating the increase of plasma cells could indicate a good outcome of AP. Decrease in *S100A8* and *S100A9* and increase in *DUSP1* in the B cells of AP patients with complications could act as candidate mechanisms by which B cells played a protective role.

The single‐cell transcriptome analysis identifies alterations in cell populations and transcriptomic differences between cells. In the present study, we detected a relatively rare cell population and defined it as “plasma cells,” which were mainly expressed in the complicated cases of AP [25]. It has been reported that plasma cells produced protective antibodies such as IgA and IgM during humoral immune responses [26]. Early antibodies such as natural IgM were produced by plasma cells and played a protective role. By contrast, the later antibody responses and cellular functions were pathogenic in conditions such as renal ischemia‐reperfusion injury [27]. It has been demonstrated that immunosuppression occurred in the early phase of complicated AP and associated with lower serum IgG and IgM titers [28]. It has also been reported that plasma levels of IgA, IgG, and IgM were increased in AP patients at day 10 compared with at admission [29]. In our study, we found that constitutive MZB1‐expressing plasma cells were significantly increased in the PBMCs of complicated group with the clinical course resolution after treatment. We also demonstrated that increased plasma cells were not only associated with increased IgA, but also correlated with increased anti‐inflammatory cytokines like IL‐10 and decreased pro‐inflammatory cytokines like TNF‐α and IL‐6. It is known that MZB1 promotes J‐chain binding to IgA and the secretion of dimeric IgA, but not IgG; mice lack of MZB1 had diminished secretion of IgA into the gut in response to acute inflammation and developed severe colitis [30]. Our results validate the findings of MZB1 and IgA in acute inflammation and suggest that increase in the numbers of plasma cells and the amounts of IgA secretion might be the prognosis indicator of recovery for complicated AP.

TCR and BCR are indispensable functional receptors of T/B cells. Their diversities ensure that T/B cells can respond to various antigens. Increased diversity of TCR/BCR has been observed in various acute critical illnesses. Patients with acute myocardial infarction had distinct TCR repertoires and *V/J* genes [31], and T‐cell clonotype diversity was tightly associated with cardiovascular condition [32]. In patients with acute myeloid leukemia, increased TCR repertoire diversity and clonal expansion have been observed [33]. In a separate note, B‐cell receptor repertoire diversity was elevated in patients with acute coronary syndrome [34]. BCR diversity in patients with acute rejection after renal transplantation was changed [35]. Therefore, changes in BCR diversity might also valuable to establish patient immune status. Here, we performed scTCR‐seq and scBCR‐seq on AP patients and found that TCR/BCR diversity was dramatically increased in complicated AP. Besides, the clonotype diversity of TCR/BCR exhibited different status during disease progression and showed the turning point at day 3, which proved that early prediction of overall complications or persistent organ failure was essential for prompt targeted interventions. The significance of CD8^+^ T cells and plasma cells in AP patients was also unraveled through integrating the omics‐level analysis. However, the mechanism and effect of TCR/BCR reconstitution are still not clear. How TCR/BCR immune repertoire modulates the inflammatory milieu during AP progression and resolution also warrants further investigations.

By using the machine learning methods, we established an accurate severity predictive model based on a nine‐gene transcriptome signature derived from PBMCs of AP patients at day 1 since admission. Besides, MZB1^+^ plasma cells were continuously increased during AP recovery, which would be helpful for the clinicians to treat AP with complications timely and establish novel therapeutic approaches in the future. However, this study has several limitations. First, despite the strategic use of key sampling time points and strict patient inclusion criteria, the sample size of our discovery cohort for single‐cell analysis remains small. Additionally, the model's reliance on RT‐qPCR infrastructure, while providing high accuracy, poses a practical consideration for its widespread clinical adoption compared to simple bedside variables. Finally, the precise biological mechanisms linking this B‐cell signature to AP pathophysiology require further elucidation. Therefore, future work will prioritize large‐scale, multi‐center prospective validation to firmly establish its clinical utility, alongside dedicated functional studies to uncover the underlying biology and potential new therapeutic targets within the B‐cell axis.

In conclusion, by integrating single‐cell transcriptomics with machine learning, we systematically profiled the peripheral immune landscape in AP. Our analysis uncovered a novel B‐cell driven, nine‐gene signature that is strongly associated with disease severity and was successfully translated into a predictive model with high diagnostic performance. From an application standpoint, this model offers a powerful new tool for rapid and accurate risk stratification, enabling clinicians to make earlier, more informed decisions to improve patient outcomes.

## 4 METHODS

### 4.1 Study Population and Blood Sample Collection

All participants enrolled in this study provided informed written consent. Two healthy volunteers were enrolled for a single collection of PBMCs. Patients diagnosed with AP as per the revised Atlanta classification criteria [6] were included from the Emergency Department. Patients were excluded if they were pregnant or lactating, or had prior chronic pancreatitis, malignant tumors, severe comorbidities of cardiovascular, respiratory, liver, and renal systems [8, 36]. In designated patient cohorts, samples of PBMCs and serum were collected at given time nodes—days 1, 3, and 7 after admission.

Persistent organ failure was defined as any organ dysfunction of respiratory, circulatory, or renal alone or in combination with individual modified Marshall score ≥ 2 lasting for more than 48 h; local complications were defined as the presence of acute necrotic collection or wall‐off necrosis, acute peripancreatic fluid collection or pancreatic pseudocyst, and extrapancreatic complications [6]. The disease severity of patients were initially defined as mild, moderately severe, and severe AP by the revised Atlanta classification [6] according to the status of organ failure and local compilations [6]. Thereafter, they were reallocated to complicated group (moderately severe and severe AP) and uncomplicated group (mild AP) for comparative analyses.

### 4.2 Mouse AP Models

Male C57BL/6 mice (8–10 weeks old, 22–25 g) were purchased from Shanghai SLAC Laboratory Animal Co. (Shanghai, China). Mice were housed in a specific pathogen‐free facility under controlled conditions (22 ± 1°C, 50%–60% humidity, 12‐h light/dark cycle) with free access to standard chow and water. And they were randomly divided into three groups (*n* = 6 per group): control, CER‐AP, and CER/LPS‐AP. Mice in the control and CER‐AP groups received intraperitoneal injection of sterile normal saline or 100 µg/kg cerulein (C9026, Sigma‐Aldrich) for 10 times at hourly intervals [37]. Mice in the CER‐AP group received an additional intraperitoneal injection of 30 mg/kg LPS (L2630, Sigma‐Aldrich) immediately at the end of the cerulein injection procedure. All mice were euthanized 12 h after the first saline/cerulein injection. Blood and pancreas were collected for FACS analysis.

### 4.3 PBMC and Serum Isolation

PBMCs of human or mice were isolated using Ficoll‐Paque Plus (No. 17‐1440‐03, GE Healthcare Pharmacia) from anticoagulated blood diluted in RPMI 1640 (No. 22400089, Gibco). The diluted blood was layered over Ficoll‐Paque Plus and centrifuged at 800 × *g* for 20 min at room temperature. PBMCs from the interphase were collected and washed for subsequent single‐cell sequencing, flow cytometry. The further stored PBMCs after washing were subjected to RT‐qPCR analysis.

Peripheral blood samples collected using serum separator tube (No. 367955, BD Biosciences) were centrifuged at 1000 × *g* to isolate serum and stored in liquid nitrogen for determining biochemical markers for relevant functional validation studies.

### 4.4 Single‐Cell RNA Sequencing

scRNA‐Seq and V(D)J libraries were prepared using the Chromium Single Cell 5' Library & Gel Bead Kit (No. 1000264, 10X Genomics) and V(D)J Enrichment Kit (No. 1000252, 10X Genomics) according to instructions from the manufacturer. Single‐cell gel bead‐in‐emulsions were generated with a target of 6000 cells per sample. After reverse transcription, barcoded cDNA was purified, amplified, and quantified using the Qubit high‐sensitivity DNA assay (No. Q33231, Thermo Fisher Scientific). Library size distribution was assessed with a Bioanalyzer 2200 (Agilent Technologies). Sequencing was performed on an Illumina platform using a 150‐bp paired‐end run.

Single‐cell sequencing data were processed using Cell Ranger 3.0.2 (10X Genomics) for alignment and barcode demultiplexing. The expression matrix was analyzed with Scanpy. Quality control filtering removed cells with fewer than 500 genes or genes detected in fewer than five cells, as well as cells with > 10% mitochondrial gene expression. Cells in the top 5% unique molecular identifier (UMI) counts were also excluded to avoid doublets, confirmed by Scrublets. Variable genes were selected for clustering. After combining all data, the top 2000 variable genes were chosen, and UMI counts and mitochondrial gene proportions were regressed out. Batch correction was performed using BBKNN with donor as batch key. Leiden clustering was applied with a resolution of 1. DEGs were identified using Scanpy's “rank_gene_groups” function with a Wilcoxon test and log fold change threshold of 0.5. GO analysis was conducted using the “gseapy” Python package. Cell–cell interaction analysis was performed with the R package CellChat [38]. Pseudotime analysis was carried out using the R package monocle2 [39] on B cells and plasma cells, with default parameters.

### 4.5 Single‐Cell TCR and BCR Sequencing

Data processing was performed using the Cell Ranger v. 3.0.2 V(D)J pipeline with the GRCh38 human genome reference. Diversity metrics, including barcode information and clonotype frequency, were obtained from Cell Ranger. The filter contig annotation output was loaded into R v. 4.0.0 and analyzed using the scRepertoire v. 1.3.2 package [40]. Clonotypes were identified based on V(D)J genes and CDR3 nucleotide sequences. Repertoire diversity was evaluated using indices implemented in scRepertoire. TCR and BCR contigs were integrated with scRNA‐seq data. The DimPlot function in Seurat v. 3.2.1 was used to visualize TCR and BCR distributions among cell types defined by scRNA‐seq. UMAP plots projected T and B cells, their subtypes, and prominent clonotypes using barcode data.

### 4.6 FACS Analysis

Human PBMCs were resuspended in FACS buffer (1% FBS in PBS) and centrifuged at 400 × *g* for 5 min. Surface staining was performed with CD45 (No. 304024, BioLegend) and CD19 (No. 302216, BioLegend) antibodies for 15 min in the dark at room temperature. After two washes, cells were sorted using a Fortessa flow cytometer (BD Biosciences). For intracellular staining, PBMCs were fixed/permeabilized (No. 554714, BD Biosciences) for 20 min at 4°C, then incubated with anti‐MZB1 (No. ab105229, Abcam), anti‐IGJ (No. 130‐114‐608, Miltenyi Biotec), and anti‐CD38 (No. 12‐0388‐42, Thermo Fisher Scientific) antibodies for 30 min in the dark. Cells were washed three times, resuspended, and sorted. Data were analyzed using FlowJo 10 software (Treestar Inc.).

PBMCs were isolated using Ficoll‐Paque Plus at 800 × *g* for 20 min at room temperature. PBMCs from the interphase were collected and washed. The pancreatic tissues were minced and digested in RPMI‐1640. Digested tissue was filtered through a 70‐µm strainer and washed with PBS + 2% FBS. Surface antibodies (CD45, CD19, CD11b, F4/80, CD8a, CD38, and Ly6C) were added and incubated for 30 min at 4°C in the dark. For MZB1, cells were fixed and permeabilized. Then MZB1 antibody was added and incubated for 45 min at 4°C. Cells were washed twice with PBS + 2% FBS, re‐suspended, and sorted. Data were analyzed using FlowJo 10 software (Treestar Inc.).

### 4.7 Serum Immunoglobulins and Cytokine Measurements

Quantification of serum immune markers including immunoglobulins (IgA [No. RK00200, Abclonal], IgG [No. RK00393, Abclonal], IgM [No. RK00097, Abclonal]) and cytokines (TNF‐α [No. RK00030, Abclonal], IL‐6 [No. RK00004, Abclonal], IL‐10 [No. RK00012, Abclonal]) was performed according to standardized ELISA protocols. Microtiter plates pre‐coated with antigen‐specific capture antibodies received diluted serum samples, followed by overnight incubation at 4°C. Sequential incubation phases included (1) washing steps to remove unbound components, (2) application of biotinylated detection antibodies, and (3) streptavidin‐HRP conjugate binding. Chromogenic reactions initiated by TMB substrate development were terminated using sulfuric acid, with optical density measurements at 450 nm acquired via microplate spectrometry. Quantitative interpretation utilized parallel‐run calibration standards with predetermined concentrations to establish reference curves [41].

### 4.8 RT‐qPCR Assays

PBMCs were resuspended in 100 µL PBS per 1 × 10^6^ cells before directly extracting total RNA. mRNA was reverse‐transcribed into cDNA using the RevertAid First Strand cDNA Synthesis Kit (No. K1622, ThermoFisher). RT‐qPCR was performed on an ABI StepOne Plus system (Applied Biosystems) using SYBR Green PCR Master Mix (No. 4309155, ThermoFisher). Primer sequences are provided in Table S2.

### 4.9 Machine Learning

“FindMarkers” function in “Seurat” package of R language was performed to obtain the DEGs of CD19^+^ B cells collected at day 1 post diagnosis in the single‐cell atlas by setting the |logFC| as 0.25 and adjusted *p* value as 0.05. PCA was carried out to identify the profile of B cells in samples of complicated group or uncomplicated group. To improve the computing efficiency, the TOP 2000 genes ranked by the variance in expression levels across cells were used as the input data. Prior to training, data preprocessing steps such as normalization and log‐transformation were applied to ensure comparability across samples. For the RT‐qPCR data, the expression matrix of nine selected genes from PBMCs was used to construct training and testing sets. Mutual information score was used to rank and select the top genes to build a random forest classifier based on their contributions. The random forest algorithm was implemented to manage high‐dimensional data and minimize overfitting by aggregating results from multiple decision trees. The AUROC was performed to detect the performance of our model. Additional performance metrics such as sensitivity, specificity, positive and negative predictive values, and positive and negative likelihood ratios were also computed to provide a comprehensive evaluation of effectiveness of the model. For medical diagnosis, these metrics should meet specific criteria: positive and negative predictive values should ideally be close to 1 (> 0.9), indicating high reliability in positive and negative predictions, respectively. Positive likelihood ratio should exceed 10 for strong diagnostic support, while negative likelihood ratio should be less than 0.1 to effectively rule out conditions [42, 43]. These standards ensure the robustness and suitability of any models for clinical applications. All models were evaluated using five‐fold cross‐validation to ensure robustness and mitigate bias. The random forest model was built using the scikit‐learn library (version 1.1.1), with default hyperparameters unless otherwise noted. Tuning was performed using grid search to optimize parameters such as the number of trees in the forest, maximum depth, and minimum samples required to split a node. The final model was selected based on the highest cross‐validated AUROC score. All statistical analyses and model training were conducted using Python (version 3.10) in a Jupyter notebook environment.

### 4.10 Statistical Analysis

Statistical analysis was performed with GraphPad Prism v. 8 (Graph Pad Software, La Jolla, CA, USA). For longitudinal comparisons within groups (e.g., temporal changes in plasma cell proportions), paired Student's *t*‐tests were applied. Between‐group comparisons (e.g., gene expression differences between complicated and uncomplicated groups) were analyzed using the non‐parametric Mann–Whitney *U* test. Categorical variables were compared via Fisher's exact test. All tests were two‐tailed, with *p* < 0.05, *p* < 0.01, *p* < 0.001 defining statistical significance.

## Author Contributions

Conceptualization: Jian Fei, Dan Xu, Wei Huang, and Ying Chen. Methodology: Jian Fei and Ying Chen. Formal analysis and investigation: Jian Fei, Rongli Xie, Guohui Xiao, Kaige Yang, and Xiaofeng Wang. Writing – original draft preparation: Dan Xu, Rongli Xie, Guohui Xiao, and Kaige Yang. Writing – review and editing: Jian Fei, Wei Huang, Kaige Yang, Xiaofeng Wang, and Cong Chen. Funding acquisition: Jian Fei, Erzhen Chen, Dan Xu, and Ying Chen. Resources: Dan Xu, Rongli Xie, Guohui Xiao, Kaige Yang, Xiaofeng Wang, Cong Chen, Dongjie Shen, Jianming Yuan, Min Ding, Tong Zhou, Erzhen Chen, Ying Chen, Wei Huang, and Jian Fei. Supervision: Jian Fei, Dan Xu, Wei Huang, Rajarshi Mukherjee, and Robert Sutton. All authors have read and approved the final manuscript.

## Ethics Statement

This study was approved by the Ruijin Hospital Ethics Committee affiliated with the Shanghai Jiao Tong University School of Medicine. APPROVAL NUMBER for clinical experiment was 2019‐90. Written informed consent was obtained from all participants. All experiments were approved by the Shanghai Jiao Tong University Institutional Animal Care and Use Committee (Ethics Approval No. SYXK2018‐0027).

## Conflicts of Interest

The authors declare no conflicts of interest.

## Supporting information




**Supplementary Figure 1**: Batch effect correction for single‐cell RNA‐seq. A. Single cells labeled by donor before batch correction. B. Single cells labeled by donor after batch correction. **Supplementary Figure 2**: Cell‐cell interaction analysis. Each panel showed strength of interaction between individual cell type such as B cell or CD14_monocyte with all other cell types. Widths of edges represent inferred interaction strengths between nodes (cell types). **Supplementary Figure 3**: FACS quantification of immune cells in AP mice. (A) Schematic workflow of flow cytometry analysis for immune cell subsets. (B‐E) Flow cytometry plots (left) and quantitative summaries (right) of immune cells (macrophages (B), Ly6C^high^ macrophages and Ly6C^low^ macrophages (C), B cells (D), plasma cells (E)) across experimental groups (Control, CER, CER+LPS). *p < 0.05; **p < 0.01; ***p < 0.001, ****p < 0.001. CER, Cerulein; LPS, lipopolysaccharide. **Supplementary Figure 4**: Single‐cell T cell receptor‐seq analysis. A. Length distribution of CDR3 sequences. B. Clonal proportion of TCR. CDR3, complementarity determining region 3; TCR, T‐cell receptor. **Supplementary Figure 5**: Single‐cell B cell receptor‐seq analysis. A. Length distribution of CDR3 sequences. B. Clonal proportion of BCR. CDR3, complementarity determining region 3; BCR, B‐cell receptor. **Supplementary Figure 6**: FACS quantification of MZB1^+^, IGJ^+^, and CD38^+^ cells in the PBMC of AP patients. A. Representative of FACS sorting quantification of MZB1^+^ cells in samples drawn from three samples in the uncomplicated group at days 1, 3, and 7. B. Representative of FACS sorting quantification of MZB1^+^ cells in samples drawn from samples of complicated group at days 1, 3, and 7. C. Representative of FACS sorting quantification of IGJ^+^ cells in samples drawn from samples of S group at days 1, 3, and 7. D. Representative of FACS sorting quantification of CD38^+^ cells in samples drawn from samples of complicated group at days 1, 3, and 7. **Supplementary Figure 7**: Detection of cytokines of patients in uncomplicated and complicated groups. A‐C, Different cytokines were measured among uncomplicated (1, 3, and 7 days) and complicated (1, 3, and 7 days) groups. *p < 0.05, **p < 0.01; ***p < 0.001, ****p < 0.001. **Supplementary Figure 8**: Gene enrichment analysis of nine gene signatures. **Supplementary Figure 9**: Expression level of 9‐gene signature in different immune cell clusters. **Supplementary Figure 10**: Metrices used to evaluate the random forest classifier in PBMC samples. A‐B. AUROC for predicting persistent organ failure. C. Table showed the sensitivity, specificity, AUROC, PPV, NPV, PLR and NLR score of the RT‐qPCR‐based machine learning model. PBMC, peripheral blood mononuclear cells; AUROC, area under the receiver operating characteristic; RT‐qPCR, real‐time quantitative reverse transcription PCR. **Supplementary Figure 11**: AUROC for external validation of prediction models. (A‐B) AUROC of train and test the model using GSE194331 datasets for complicated acute pancreatitis. (C‐D) AUROC of train and test the model using GSE194331 datasets for persistent organ failure. AUROC, area under the receiver operating characteristic. **Supplementary Figure 12**: Macro‐AUROC for external validation of prediction models. (A‐B) Macro‐AUROC of the model in the training (A) and testing cohorts (B) using the GSE194331 and GSE65682 datasets respectively for identifying complicated acute pancreatitis. (C‐D) Macro‐AUROC of the model in the training and testing cohorts using the GSE194331 and GSE65682 datasets for predicting persistent organ failure. AUROC, area under the receiver operating characteristic curve. **Supplementary Table 1**: Baseline characteristics and clinical outcomes of 114 patients for RT‐qPCR validating machine learning‐selected 9 genes. **Supplementary Table 2**: Primer sequences used for RT‐qPCR. **Supplementary Table 3**: One hundred training experiments for predicting complicated AP.

## Data Availability

Raw sequencing data were submitted to the Genome Sequence Archive (GSA) for Human (accession number HRA011687) at the National Genomics Data Center (NGDC), China National Center for Bioinformation (CNCB) [44, 45].
