# Correction to “Single‐Cell Transcriptomic Atlas of Peripheral Blood Reveals B‐Cell‐Driven Signature Predictive of Acute Pancreatitis Severity”

**DOI:** 10.1002/mco2.70955

**Published:** 2026-08-20

R. Xie, G. Xiao, K. Yang, et al. “ Single‐Cell Transcriptomic Atlas of Peripheral Blood Reveals B‐Cell‐Driven Signature Predictive of Acute Pancreatitis Severity.” MedComm 6, no. 10 (2025): 6, e70350. https://doi.org/10.1002/mco2.70350.

The image in Figure S3 is misplaced. The correct results should be as shown below. The authors declare that these corrections do not affect the description, interpretation, or conclusions detailed in the original manuscript.


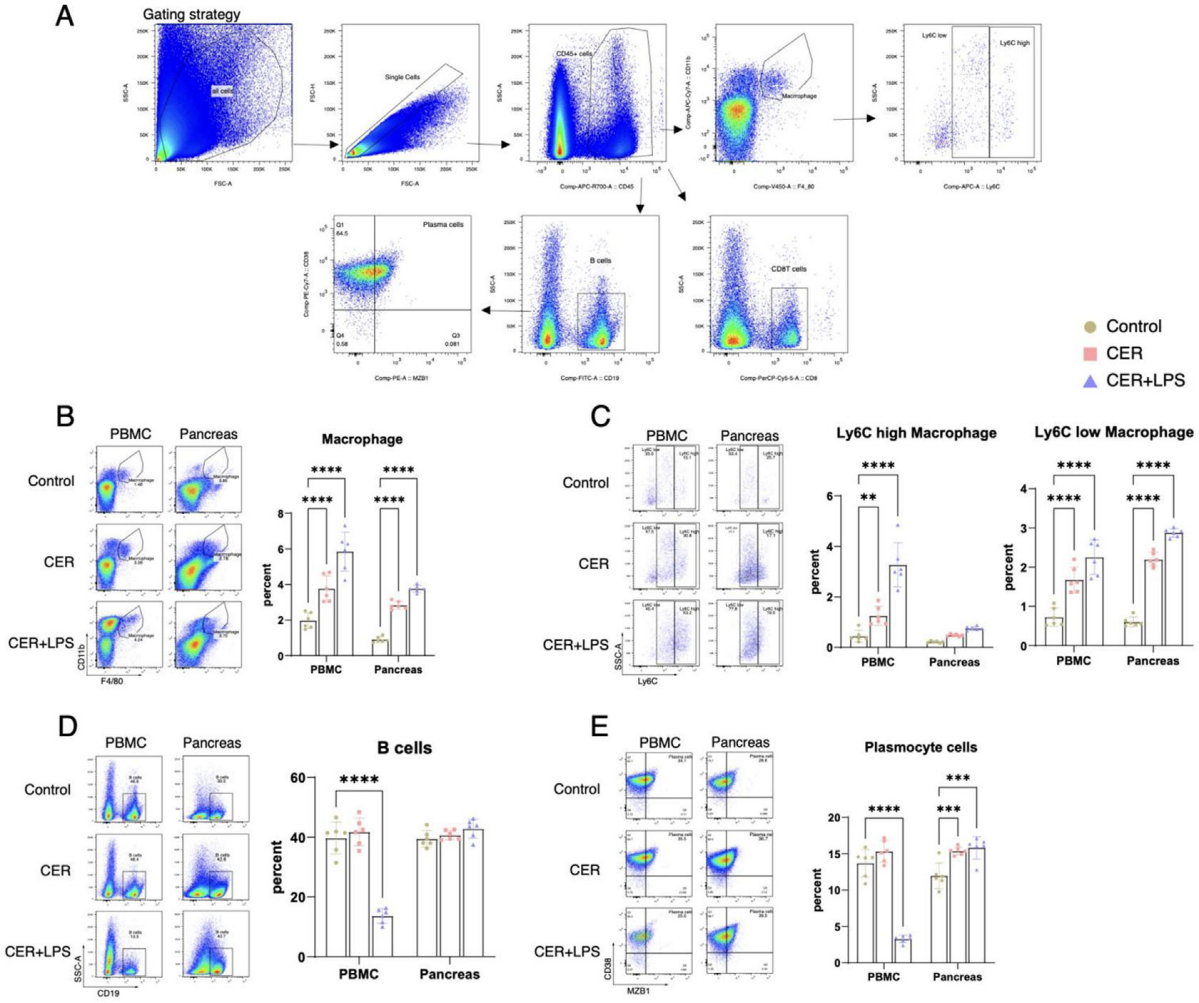


We apologize for this error.

