# Supplementary material for: Single‐Cell Transcriptomic Atlas of Peripheral Blood Reveals B‐Cell‐Driven Signature Predictive of Acute Pancreatitis Severity
Source: MedComm (2020). 2025 Sep 14;6(10):e70350. doi: 10.1002/mco2.70350 (PMC12434316; doi:10.1002/mco2.70350)
Supplement: Supplementary file 1 — Supplementary Figure 1: Batch effect correction for single‐cell RNA‐seq. A. Single cells labeled by donor before batch correction. B. Single cells labeled by donor after batch correction. Supplementary Figure 2: Cell‐cell interaction analysis. Each panel showed strength of interaction between individual cell type such as B cell or CD14_monocyte with all other cell types. Widths of edges represent inferred interaction strengths between nodes (cell types). Supplementary Figure 3: FACS quantification of immune cells in AP mice. (A) Schematic workflow of flow cytometry analysis for immune cell subsets. (B‐E) Flow cytometry plots (left) and quantitative summaries (right) of immune cells (macrophages (B), Ly6Chigh macrophages and Ly6Clow macrophages (C), B cells (D), plasma cells (E)) across experimental groups (Control, CER, CER+LPS). *p < 0.05; **p < 0.01; ***p < 0.001, ****p < 0.001. CER, Cerulein; LPS, lipopolysaccharide. Supplementary Figure 4: Single‐cell T cell receptor‐seq analysis. A. Length distribution of CDR3 sequences. B. Clonal proportion of TCR. CDR3, complementarity determining region 3; TCR, T‐cell receptor. Supplementary Figure 5: Single‐cell B cell receptor‐seq analysis. A. Length distribution of CDR3 sequences. B. Clonal proportion of BCR. CDR3, complementarity determining region 3; BCR, B‐cell receptor. Supplementary Figure 6: FACS quantification of MZB1+, IGJ+, and CD38+ cells in the PBMC of AP patients. A. Representative of FACS sorting quantification of MZB1+ cells in samples drawn from three samples in the uncomplicated group at days 1, 3, and 7. B. Representative of FACS sorting quantification of MZB1+ cells in samples drawn from samples of complicated group at days 1, 3, and 7. C. Representative of FACS sorting quantification of IGJ+ cells in samples drawn from samples of S group at days 1, 3, and 7. D. Representative of FACS sorting quantification of CD38+ cells in samples drawn from samples of complicated group at days 1, 3, and 7. Supplementa [file MCO2-6-e70350-s001.pdf]

Supplementary Figures and Figure Legends

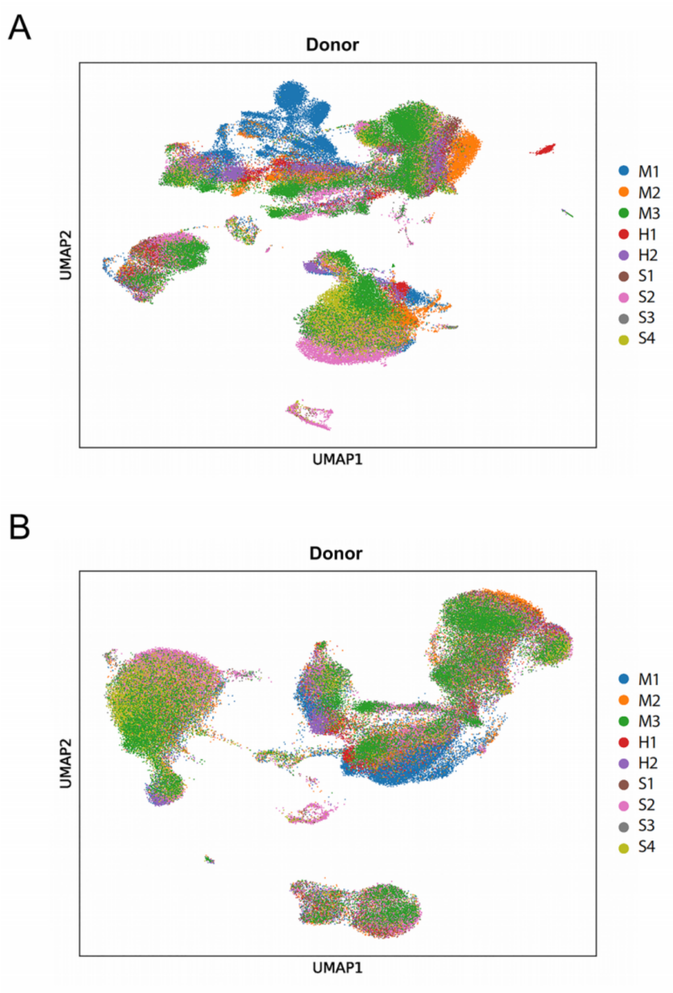

Supplementary Figure 1. Batch effect correction for single-cell RNA-seq. A. Single cells labeled by donor before batch correction. B. Single cells labeled by donor after batch correction.

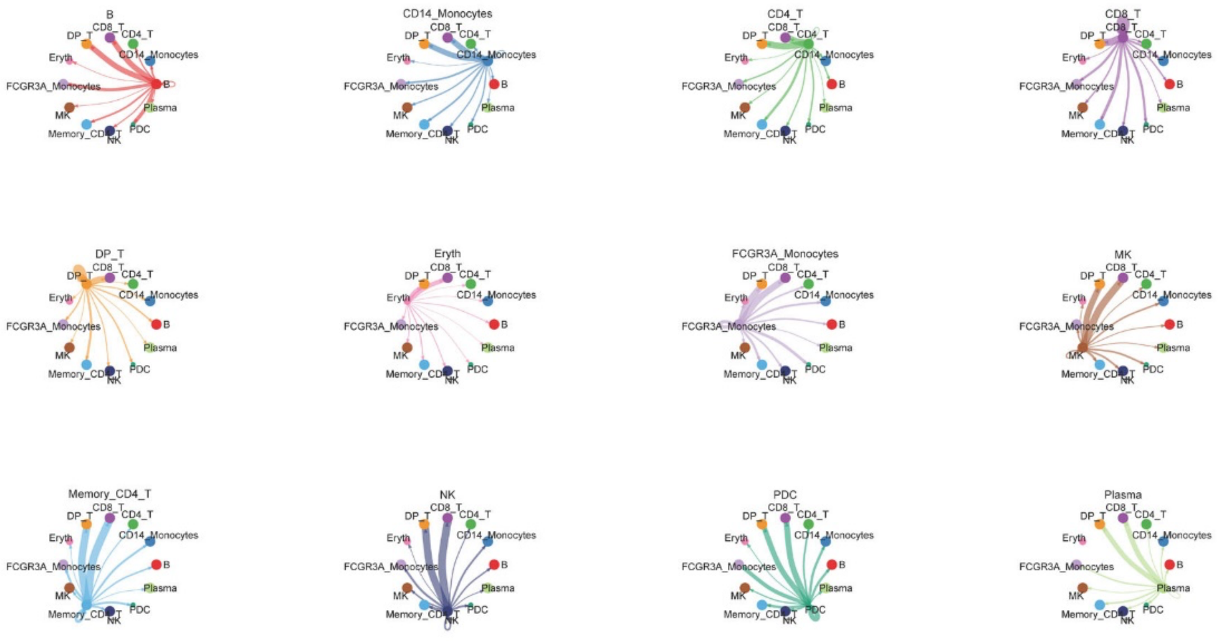

Supplementary Figure 2. Cell-cell interaction analysis. Each panel showed strength of interaction between individual cell type such as B cell or CD14_monocyte with all other cell types. Widths of edges represent inferred interaction strengths between nodes (cell types).

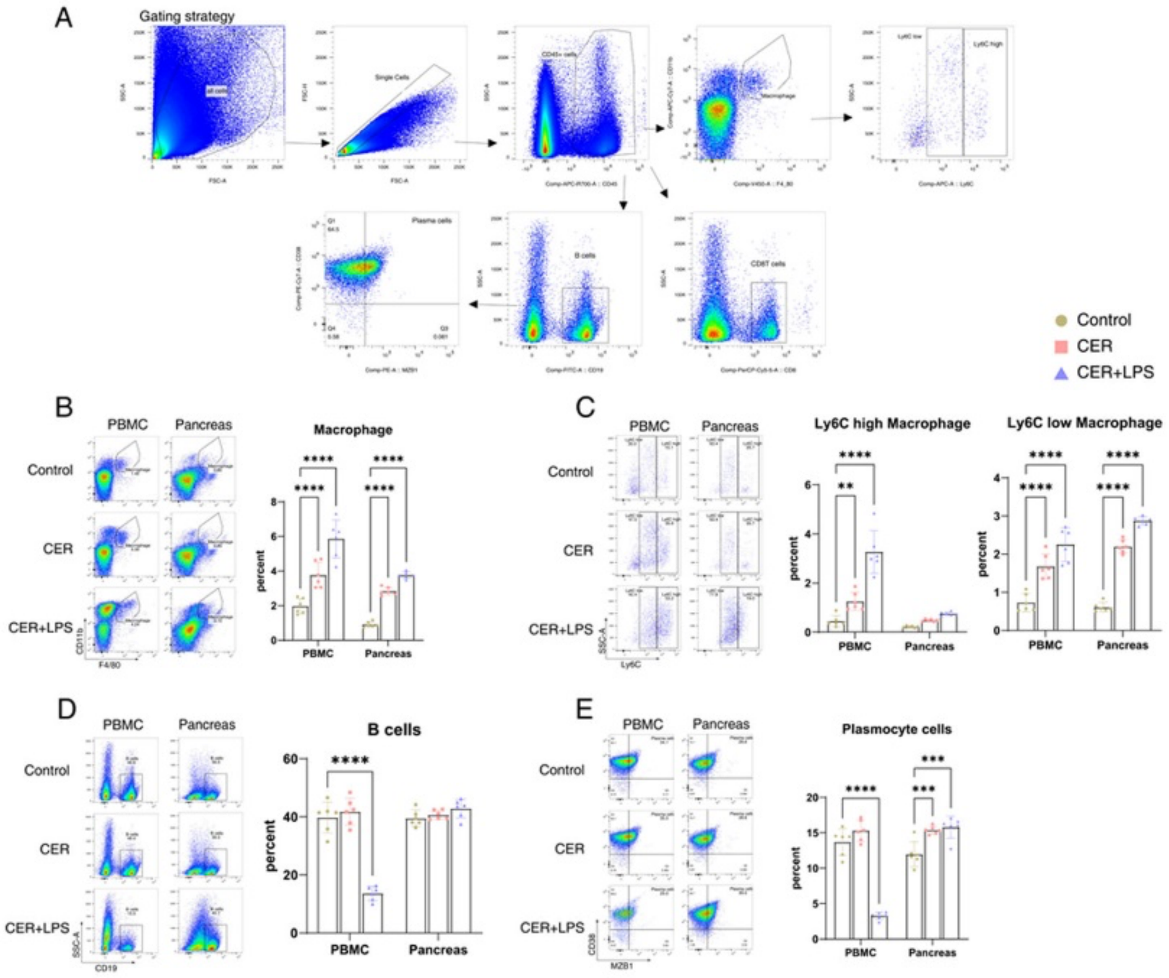

Supplementary Figure 3. FACS quantification of immune cells in AP mice. (A) Schematic workflow of flow cytometry analysis for immune cell subsets. (B-E) Flow cytometry plots (left) and quantitative summaries (right) of immune cells (macrophages (B), Ly6C^{high} macrophages and Ly6C^{low} macrophages (C), B cells (D), plasma cells (E)) across experimental groups (Control, CER, CER+LPS). **p* < 0.05; ***p* < 0.01; ****p* < 0.001, *****p* < 0.001. CER, Cerulein; LPS, lipopolysaccharide.

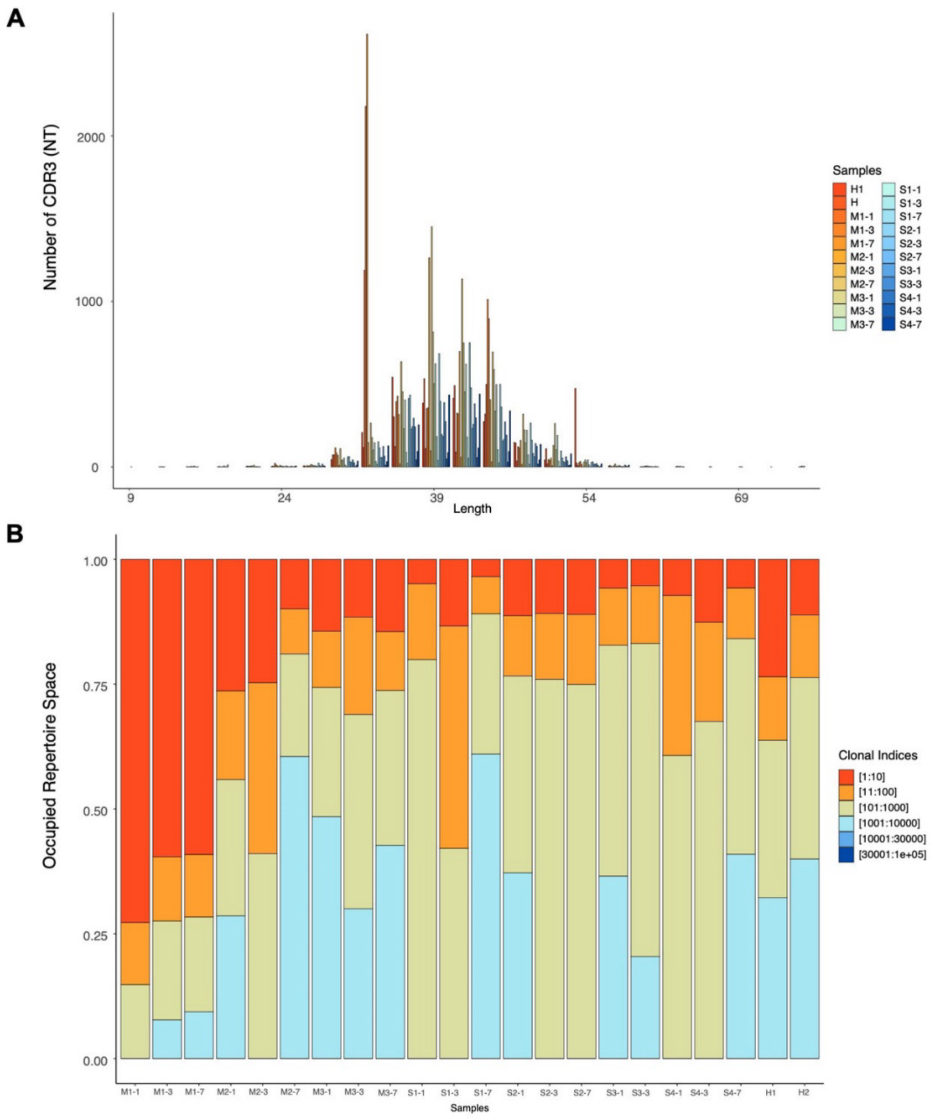

Supplementary Figure 4. Single-cell T cell receptor-seq analysis. A. Length distribution of CDR3 sequences. B. Clonal proportion of TCR. CDR3, complementarity determining region 3; TCR, T-cell receptor.

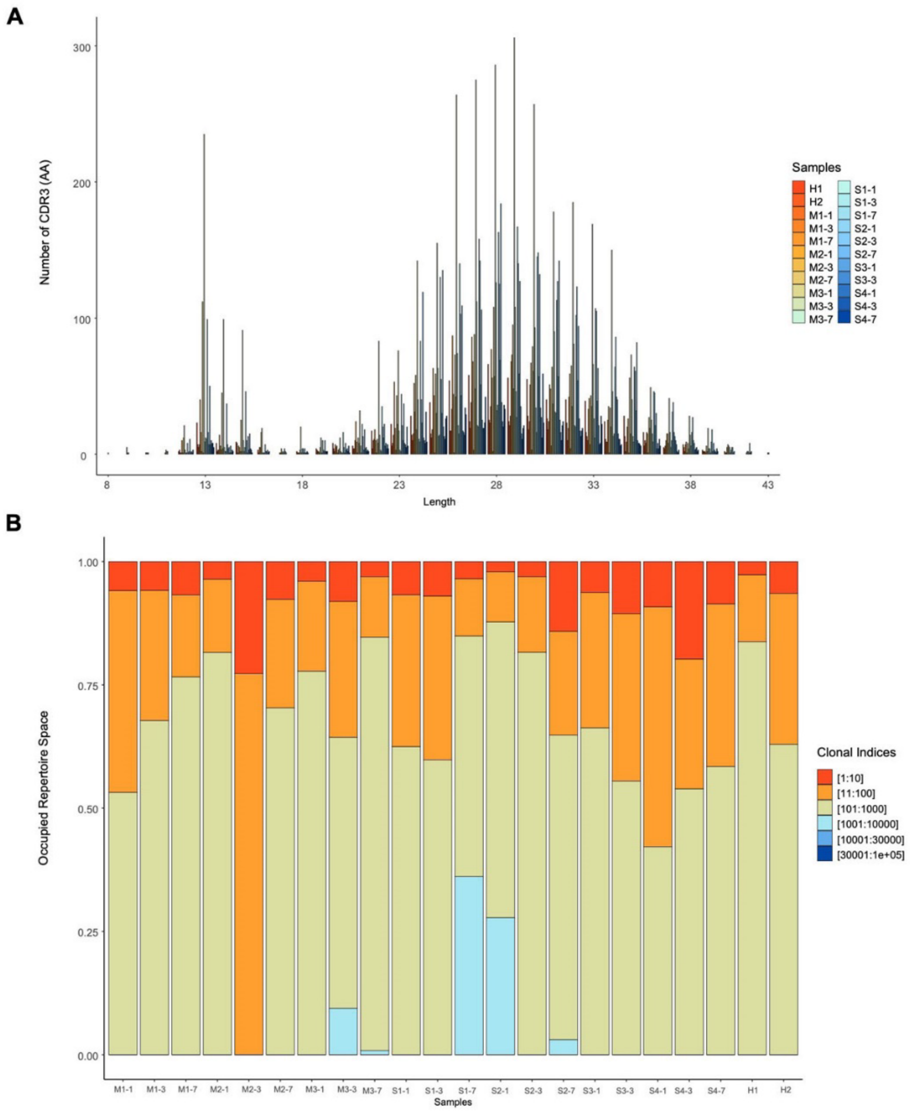

Supplementary Figure 5. Single-cell B cell receptor-seq analysis. A. Length distribution of CDR3 sequences. B. Clonal proportion of BCR. CDR3, complementarity determining region 3; BCR, B-cell receptor.

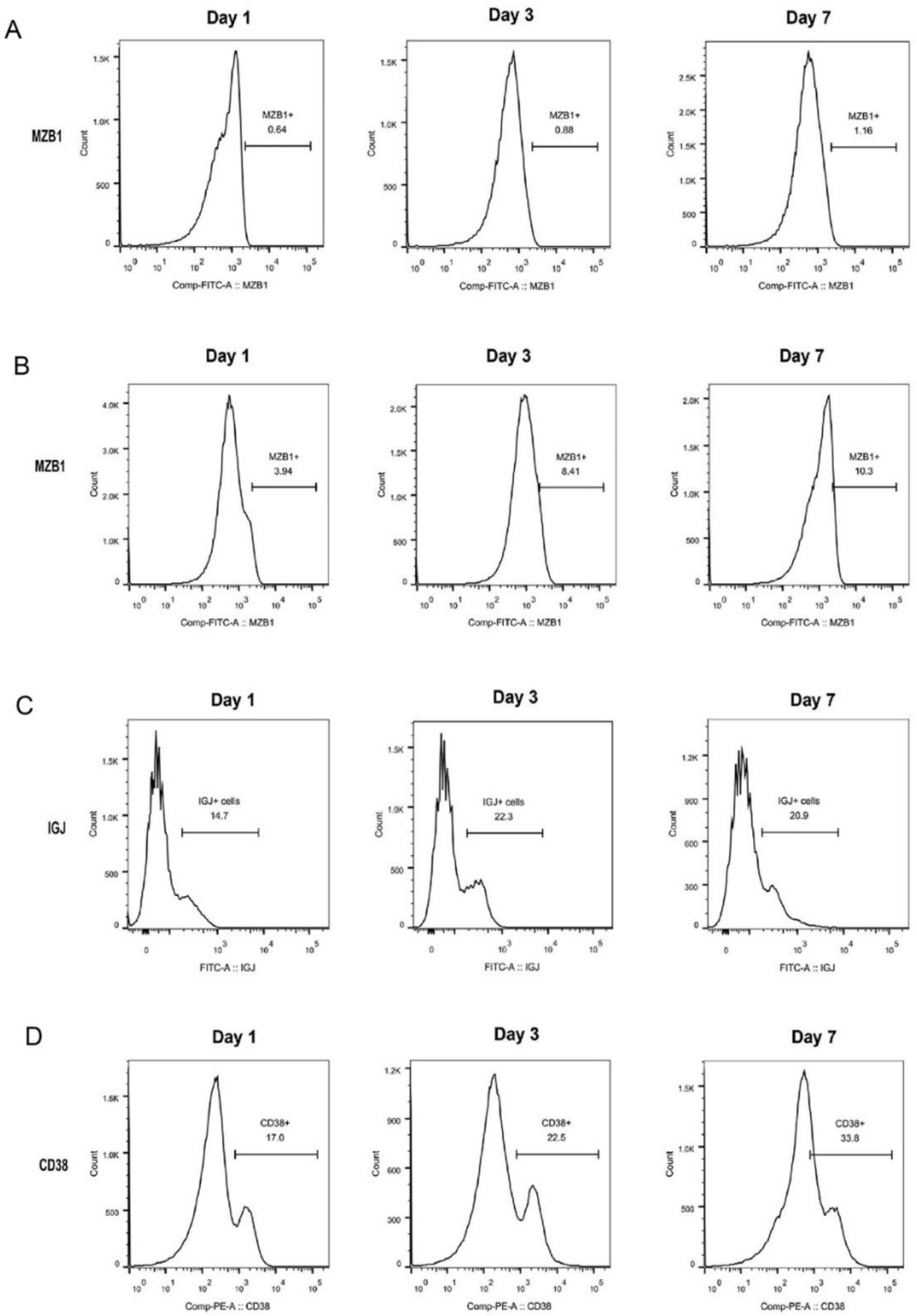

Supplementary Figure 6. FACS quantification of MZB1^{+}, IGJ^{+}, and CD38^{+} cells in the PBMC of AP patients. A. Representative of FACS sorting quantification of MZB1^{+} cells in samples drawn from three samples in the uncomplicated group at days 1, 3, and 7. B. Representative of FACS sorting quantification of MZB1^{+} cells in samples drawn from samples of complicated group at days 1, 3, and 7. C.

Representative of FACS sorting quantification of IGJ+ cells in samples drawn from samples of S group at days 1, 3, and 7. D. Representative of FACS sorting quantification of CD38+ cells in samples drawn from samples of complicated group at days 1, 3, and 7.

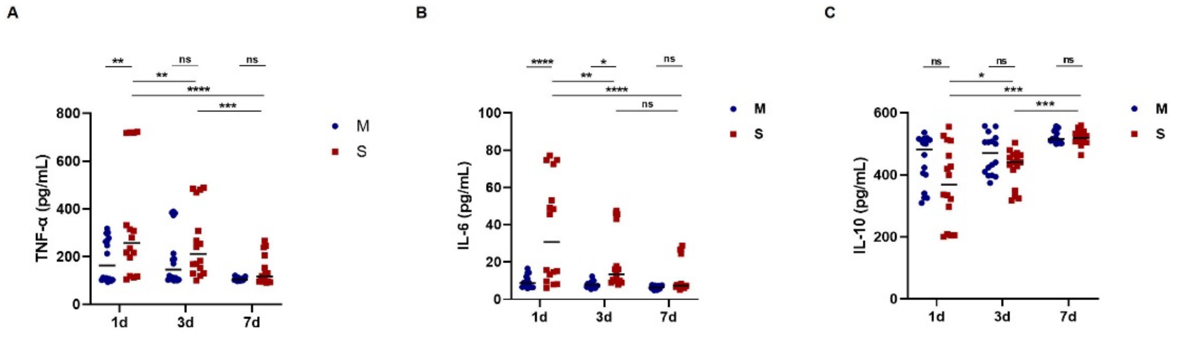

Supplementary Figure 7. Detection of cytokines of patients in uncomplicated and complicated groups. A-C, Different cytokines were measured among uncomplicated (1, 3, and 7 days) and complicated (1, 3, and 7 days) groups. *$p < 0.05$, **$p < 0.01$; ***$p < 0.001$, ****$p < 0.001$.

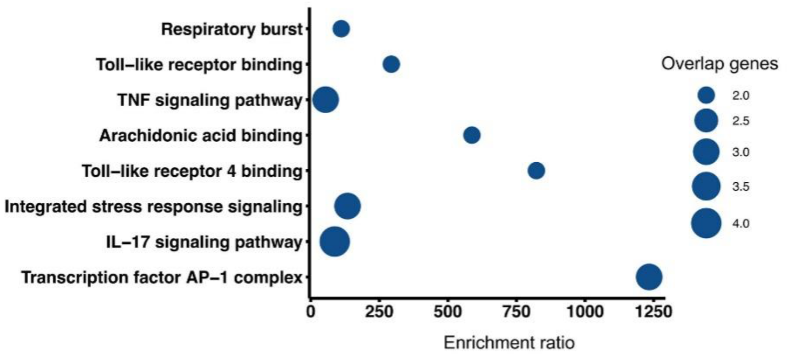

Supplementary Figure 8. Gene enrichment analysis of nine gene signatures.

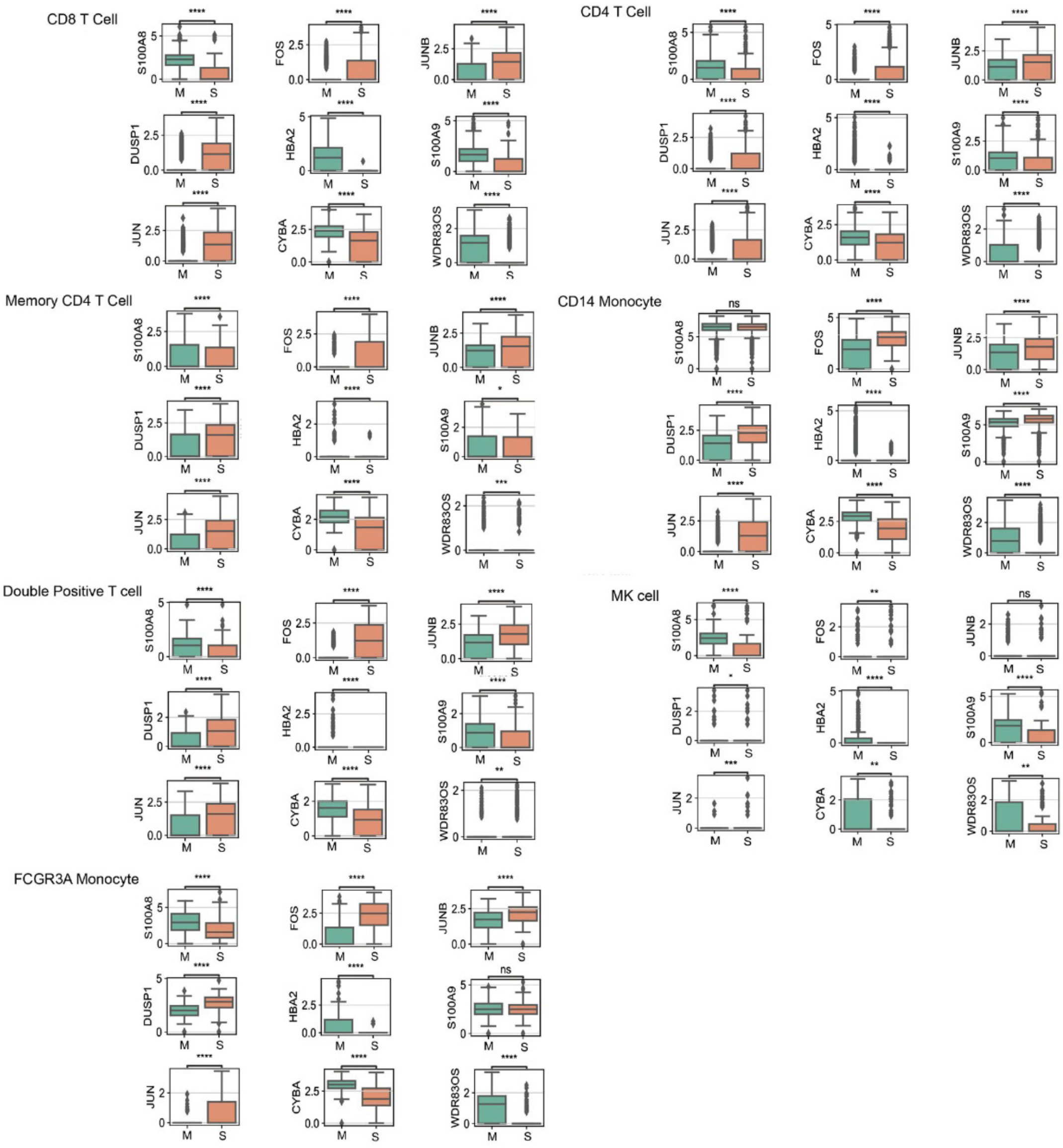

Supplementary Figure 9. Expression level of 9-gene signature in different immune cell clusters.

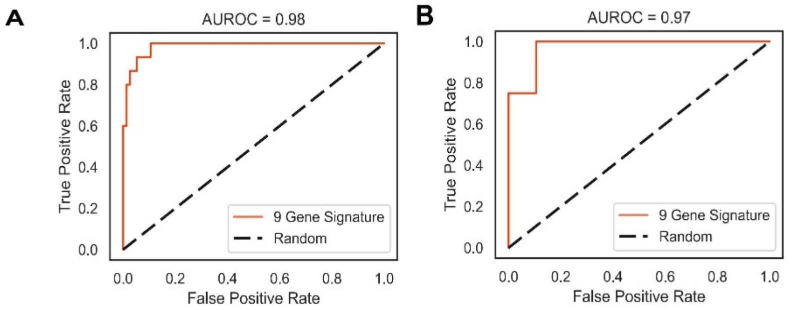

C

Metrics	Sensitivity	Specificity	AUROC	PPV	NPV	PLR	NLR
**ML training**	0.800	0.987	0.98	0.923	0.962	60.6	0.203
**ML testing**	0.750	1.000	0.97	1.000	0.95	8.25	0.250

Supplementary Figure 10. Metrices used to evaluate the random forest classifier in PBMC samples. A-B. AUROC for predicting persistent organ failure. C. Table showed the sensitivity, specificity, AUROC, PPV, NPV, PLR and NLR score of the qRT-PCR-based machine learning model. PBMC, peripheral blood mononuclear cells; AUROC, area under the receiver operating characteristic; qRT-PCR, real-time quantitative reverse transcription PCR.

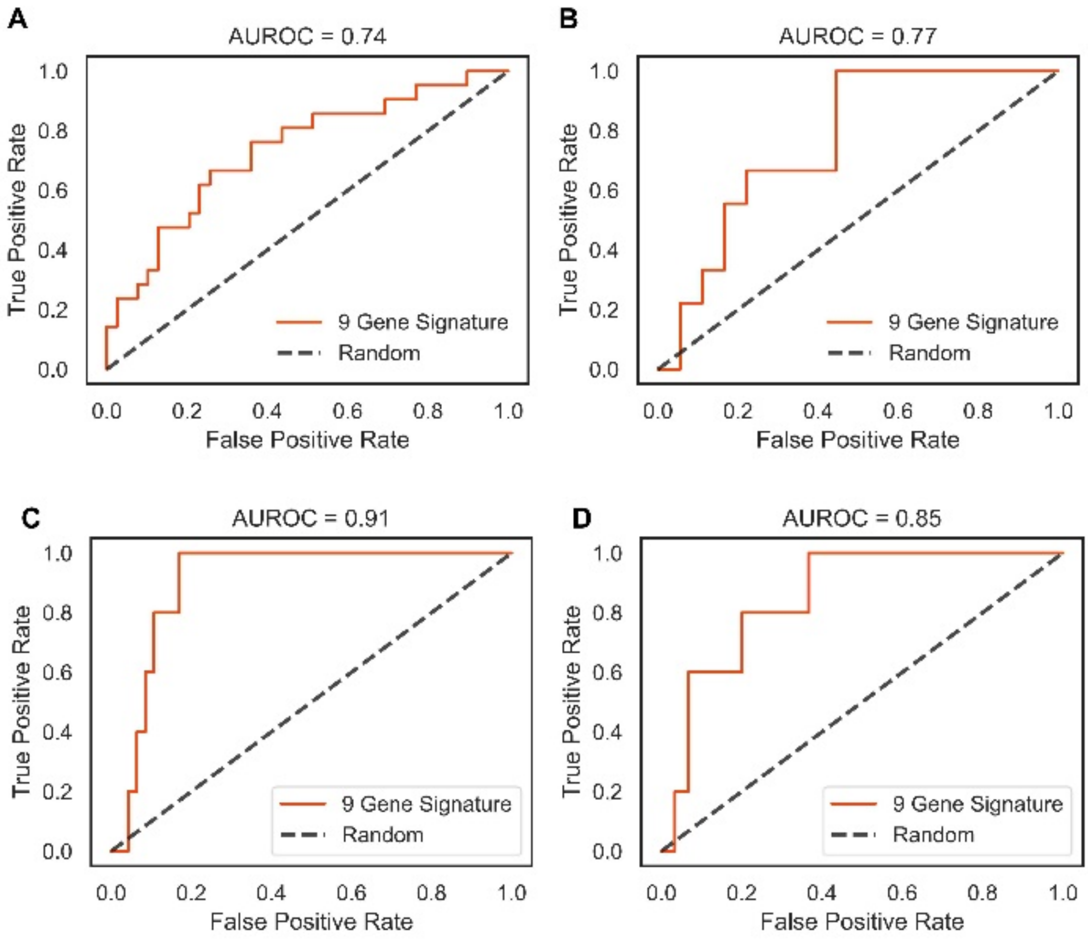

Supplementary Figure 11. AUROC for external validation of prediction models. (A-B) AUROC of train and test the model using GSE194331 datasets for complicated acute pancreatitis. (C-D) AUROC of train and test the model using GSE194331 datasets for persistent organ failure. AUROC, area under the receiver operating characteristic.

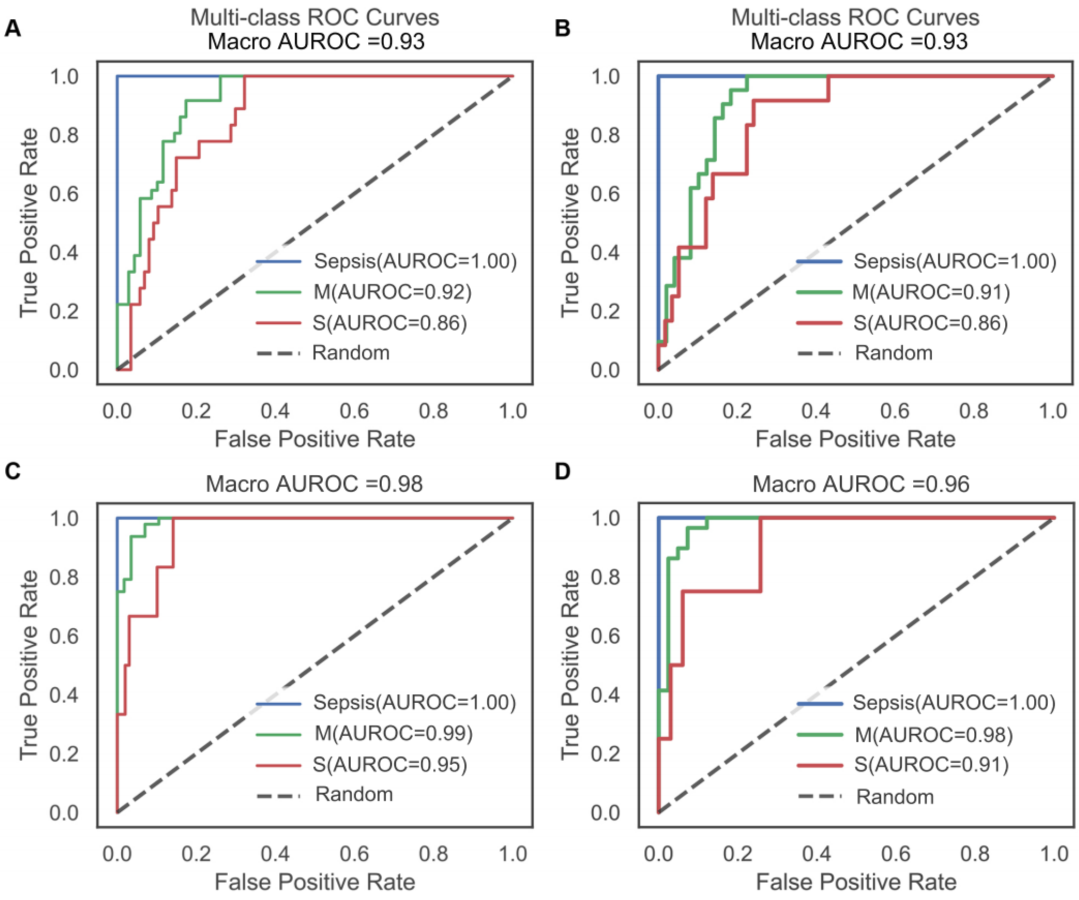

Supplementary Figure 12. Macro-AUROC for external validation of prediction models. (A-B) Macro-AUROC of the model in the training (A) and testing cohorts (B) using the GSE194331 and GSE65682 datasets respectively for identifying complicated acute pancreatitis. (C-D) Macro-AUROC of the model in the training and testing cohorts using the GSE194331 and GSE65682 datasets for predicting persistent organ failure. AUROC, area under the receiver operating characteristic curve.

Supplementary Table 1. Baseline characteristics and clinical outcomes of 114 patients for RT-qPCR validating machine learning-selected 9 genes.

	Discovery cohort (N=91)	Validation cohort (N=23)	*p*-value
**Age, years (mean±SD)**	46.9 (15.0)	49.0 (16.2)	0.554
**Gender, n(%)**			0.594
Female	34 (37.4%)	10 (43.5%)	
Male	57 (62.6%)	13 (56.5%)	
**Charlson comorbidity score**	1.7 (1.7)	1.9 (1.4)	0.748
**Etiology, n (%)**			0.268
Biliary	61 (67.0%)	18 (78.3%)	
Hyperlipidemia	21 (23.1%)	4 (17.4%)	
Alcohol	9 (9.9%)	1 (4.3%)	
**Biochemical indices on day 1, (mean±SD)**			
C-reactive protein, mg/L	145.8 (85.9)	171.9 (108.3)	0.226
Procalcitonin, ng/mL	3.3 (10.2)	2.4 (4.8)	0.695
Hematocrit, %	42.8 (7.3)	43.1 (6.0)	0.884
Hemoglobin, g/dL	140.5 (24.5)	148.0 (26.1)	0.202
White blood cell, 10^9/L	14.0 (5.6)	13.4 (4.1)	0.613
Neutrophils, %	84.6 (7.9)	84.7 (6.7)	0.962
Lymphocytes, %	13.8 (10.9)	12.5 (6.7)	0.583
Platelets, 10^9/L	203.4 (77.1)	190.0 (67.5)	0.448
Calcium, mmol/L	2.0 (0.3)	2.2 (0.6)	0.116
Lactate, mmol/L	2.7 (2.7)	2.6 (1.3)	0.956
Albumin, g/dL	37.2 (6.7)	38.8 (8.9)	0.338
Creatinine, mg/dL	0.9 (0.8)	1.0 (0.9)	0.473
**Severity, n(%)**			
Uncomplicated	31 (34.1%)	8 (34.8%)	0.949
Complicated	60 (65.9%)	15 (65.2%)	
Persistent organ failure	22 (24.2%)	6 (26.1%)	0.765
**Outcome, n(%)**			0.293
Survival	90 (98.9%)	22 (95.7%)	
Death	1 (1.1%)	1 (4.3%)	
**Number of days as inpatients**	24.8 (24.8)	27.2 (21.6)	0.670

Supplementary Table 2. Primer sequences used for qRT-PCR.

Gene	Primer	Sequence
S100A8	Forward primer	GAGACCGAGTGTCCTCAGTATATC
	Reverse primer	GCTGCCACGCCCATCTTTA
HBA2	Forward primer	GCCGACAAGACCAACGTCAAGG
	Reverse primer	AGGTCGAAGTGCGGGAAGTAGG
CYBA	Forward primer	CCCAGTGGTACTTTGGTGCC
	Reverse primer	CGGTCATGTACTTCTGTCCC
S100A9	Forward primer	ACATCATGGAGGACCTGGACAC
	Reverse primer	GGTTAGCCTCGCCATCAGCAT
WDR83OS	Forward primer	GTGTGCTTGGGTCGCTGTCTAC
	Reverse primer	AGATAGGACATCACCACGGCAGAG
DUSP1	Forward primer	AGTACCCCACTCTACGATCAGG
	Reverse primer	GAAGCGTGATACGCACTGC
JUN	Forward primer	ACGACTCATACACAGCTACGG
	Reverse primer	GCTCGGTTTCAGGAGTTTGTAGT
FOS	Forward primer	CCGGGGATAGCCTCTCTTACT
	Reverse primer	CCAGGTCCGTGCAGAAGTC
JUNB	Forward primer	ACGACTCATACACAGCTACGG
	Reverse primer	GCTCGGTTTCAGGAGTTTGTAGT
GAPDH	Forward primer	AGATCATCAGCAATGCCTCCT
	Reverse primer	TGAGTCCTTCCACGATACCAA

Supplementary Table 3. One hundred training experiments for predicting complicated AP.

	Precision	Recall	AUROC	F1	Accuracy Score
0	0.857064	0.830566	0.938644	0.841138	0.861111
1	0.848564	0.846982	0.93684	0.847761	0.862745
2	0.841544	0.823658	0.92176	0.831248	0.851307
3	0.83015	0.803472	0.917393	0.813835	0.837971
4	0.846375	0.82144	0.92003	0.831439	0.8527
5	0.84948	0.831392	0.929434	0.839075	0.857843
6	0.850775	0.817045	0.919945	0.82972	0.852941
7	0.840046	0.825903	0.925654	0.832099	0.851307
8	0.863402	0.846386	0.928981	0.853756	0.870704
9	0.844905	0.81907	0.937749	0.829339	0.851064
10	0.838516	0.811415	0.914564	0.821975	0.844771
11	0.825595	0.810314	0.922894	0.816879	0.838235
12	0.848363	0.832014	0.937514	0.839075	0.857843
13	0.861942	0.844017	0.934674	0.851717	0.869067
14	0.860245	0.830059	0.938104	0.841827	0.86252
15	0.862378	0.836533	0.929941	0.846948	0.866013
16	0.830945	0.80707	0.925784	0.816598	0.839869
17	0.862084	0.84411	0.935735	0.851836	0.869281
18	0.855735	0.843756	0.943406	0.849141	0.865794
19	0.832211	0.81394	0.916179	0.821606	0.842881
20	0.842677	0.823066	0.929534	0.831248	0.851307
21	0.862127	0.836002	0.935806	0.846541	0.866013
22	0.833911	0.816425	0.91403	0.82383	0.844771
23	0.855622	0.831049	0.941931	0.841014	0.860884
24	0.832338	0.818549	0.918205	0.824577	0.844517
25	0.851725	0.824599	0.934098	0.835313	0.856209
26	0.825455	0.805699	0.909031	0.813839	0.836601
27	0.84536	0.827274	0.926972	0.834957	0.854575
28	0.857223	0.821831	0.931321	0.835042	0.85761
29	0.847444	0.833027	0.932583	0.839349	0.85761
30	0.83004	0.819033	0.923756	0.823959	0.843137
31	0.845339	0.811059	0.931729	0.823798	0.848039
32	0.827768	0.806946	0.914456	0.81546	0.838235
33	0.861764	0.853235	0.942826	0.857192	0.87234
34	0.852343	0.82282	0.929248	0.834295	0.855974
35	0.838063	0.820566	0.925501	0.827977	0.848039
36	0.854371	0.841492	0.940705	0.847239	0.864379
37	0.827047	0.803453	0.91315	0.812855	0.836601
38	0.857985	0.845006	0.935812	0.850798	0.86743

39	0.840068	0.81657	0.926084	0.826051	0.847791
40	0.828775	0.828042	0.924752	0.828406	0.844771
41	0.84315	0.813304	0.920773	0.824774	0.848039
42	0.851514	0.832138	0.934884	0.840316	0.859477
43	0.85951	0.834668	0.929905	0.844755	0.864157
44	0.851641	0.812352	0.928169	0.826475	0.851064
45	0.850956	0.848585	0.929888	0.849744	0.864379
46	0.86017	0.826648	0.937408	0.839404	0.861111
47	0.82276	0.815928	0.915247	0.81911	0.838235
48	0.866725	0.820972	0.933306	0.836972	0.860884
49	0.837567	0.819929	0.930278	0.827402	0.847791
50	0.855635	0.828208	0.934735	0.839056	0.859477
51	0.849248	0.840121	0.934447	0.844318	0.861111
52	0.849471	0.826276	0.927433	0.835749	0.856209
53	0.833748	0.816309	0.915095	0.82369	0.844517
54	0.832989	0.81282	0.922909	0.821145	0.842881
55	0.851048	0.817665	0.928337	0.830193	0.852941
56	0.824721	0.806822	0.918332	0.81432	0.836601
57	0.846862	0.829644	0.933744	0.837019	0.856209
58	0.858342	0.854224	0.942068	0.856207	0.870704
59	0.850244	0.809982	0.92356	0.824316	0.849427
60	0.843529	0.821958	0.938662	0.830813	0.851307
61	0.855874	0.843862	0.939275	0.849265	0.866013
62	0.820064	0.795098	0.918687	0.804847	0.830065
63	0.860256	0.846256	0.928075	0.852458	0.869067
64	0.860556	0.833549	0.922932	0.844342	0.864157
65	0.864211	0.842358	0.944463	0.851456	0.869281
66	0.834729	0.819917	0.922811	0.826346	0.846405
67	0.83163	0.810562	0.922575	0.819188	0.841503
68	0.840992	0.81545	0.919603	0.825588	0.847791
69	0.855193	0.835658	0.928169	0.843905	0.86252
70	0.854167	0.847618	0.942866	0.8507	0.866013
71	0.857588	0.825401	0.930222	0.83774	0.859477
72	0.821178	0.80782	0.920377	0.813648	0.834967
73	0.851578	0.805373	0.922251	0.8211	0.847791
74	0.843667	0.829408	0.923252	0.835656	0.854337
75	0.835179	0.811274	0.930537	0.820817	0.843137
76	0.817754	0.798466	0.915738	0.806393	0.830065
77	0.852406	0.831015	0.935788	0.839902	0.859477
78	0.852822	0.843626	0.93721	0.847855	0.864157
79	0.850784	0.828549	0.923217	0.837701	0.85761
80	0.828932	0.806415	0.928184	0.81546	0.838235
81	0.849105	0.835506	0.93463	0.841521	0.859477
82	0.869676	0.839743	0.942342	0.851558	0.870915

83	0.844442	0.815581	0.919988	0.826763	0.849427
84	0.833894	0.820918	0.920486	0.826638	0.846154
85	0.829576	0.825825	0.920342	0.827629	0.844771
86	0.868079	0.830388	0.930647	0.84442	0.866013
87	0.861092	0.837125	0.941119	0.846948	0.866013
88	0.837686	0.81532	0.911422	0.824412	0.846154
89	0.838356	0.823418	0.929899	0.829904	0.849427
90	0.859175	0.849009	0.938998	0.853652	0.869281
91	0.861217	0.845233	0.932515	0.85221	0.869281
92	0.847593	0.833136	0.918368	0.839479	0.857843
93	0.828089	0.797613	0.914805	0.80903	0.834697
94	0.859807	0.816102	0.933578	0.831465	0.855974
95	0.876585	0.860118	0.942005	0.86732	0.882353
96	0.846065	0.818044	0.924354	0.82901	0.851307
97	0.853645	0.842615	0.946106	0.847613	0.864379
98	0.81285	0.791232	0.912014	0.799865	0.824877
99	0.83654	0.808341	0.919165	0.819231	0.842881
